# Extracellular Vesicles From Prostate Cancer‐Corrupted Osteoclasts Drive a Chain Reaction of Inflammatory Osteolysis and Tumour Progression at the Bone Metastatic Site

**DOI:** 10.1002/jev2.70091

**Published:** 2025-06-23

**Authors:** Takaaki Tamura, Tomofumi Yamamoto, Akiko Kogure, Yusuke Yoshioka, Yusuke Yamamoto, Shinichi Sakamoto, Tomohiko Ichikawa, Takahiro Ochiya

**Affiliations:** ^1^ Department of Molecular and Cellular Medicine Institute of Medical Science Tokyo Medical University Tokyo Japan; ^2^ Department of Urology Graduate School of Medicine Chiba University Chiba Japan; ^3^ Laboratory of Integrative Oncology National Cancer Center Research Institute Tokyo Japan; ^4^ Division of Biological Chemistry and Biologicals National Institute of Health Sciences Tokyo Japan

**Keywords:** bone metastasis, extracellular vesicles, IL‐1β, osteoclast, prostate cancer

## Abstract

Advanced‐stage prostate cancer (PCa) frequently causes bone metastases, resulting in a poor prognosis and a 5‐year survival rate of 30%. PCa bone metastasis is a highly complex and fluctuating process, comprising of osteolytic (bone‐degrading) and osteogenic (bone‐forming) lesions. Although this system is mainly controlled by alterations in the receptor activator of NF‐κB ligand (RANKL), RANKL‐based treatment does not prolong the overall survival of patients with PCa bone metastasis. Therefore, it is essential to understand the other interactions between tumour cells and bone‐resident cells in the metastatic niche to develop novel treatments. Extracellular vesicles (EVs) play key roles in intercellular communication and actively function in the bone microenvironment. We report that PCa cells corrupt osteoclasts (OCs) via their secretomes, inducing a pathological phenotype. EVs from pathological OCs activate bone‐resorbing OCs and suppress bone‐forming osteoblasts (OBs), leading to bone destruction. Pathological OCs increased IL‐1β secretion and produced EVs with miR‐5112 and miR‐1963, targeting *Parp1* in OCs and *Hoxa1* in OBs. This led to OC maturation and IL‐1β secretion, and inhibited OB mineralization. Injection of these miRNAs in vivo promoted PCa metastasis‐disrupting bone. We report the mediation of EVs from OCs under pathological conditions that modulate the bone metastatic niche independently of RANKL.

## Graphical abstract

Graphical abstract

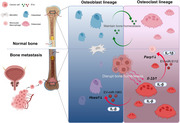

## 1 Introduction

Prostate cancer (PCa) has a high incidence worldwide, particularly in the United States of America (Bray et al. 2024; Siegel et al. 2023). PCa is one of the most frequently diagnosed cancers among men and the second leading cause of cancer‐related deaths (Siegel et al. 2023). More than 80% of patients with advanced‐stage PCa eventually develop bone metastases, which reduce the 5‐year survival rate to 30% (Sturge et al. 2011). Common sites for metastasis include the spine, ribs, pelvis and femur, which can lead to many complications and morbidities, including severe bone pain, hypercalcemia and pathologic fractures, leading to prolonged hospital stay. Moreover, these events have been correlated with reduced quality of life and mortality in patients with PCa (Nørgaard et al. 2010).

Bone is a dynamic biological system that is constantly renewed by bone‐resident cells, mainly bone‐resorbing osteoclast (OC) lineage cells and bone‐forming osteoblast (OB) lineage cells. OCs originate from monocytes, undergoing differentiation and fusion during maturation to facilitate the resorption of bone tissue. OBs, on the other hand, are derived from bone marrow mesenchymal stem cells (BM‐MSCs) and mature into osteocytes, which are the predominant cell type found in bone tissue (Roodman 2004). During physiological bone homeostasis, the activity of these two cell lineages is tightly regulated by various mediators. Notably, three molecules in the tumour necrosis factor superfamily, including the receptor activator of NF‐κB (RANK), its ligand RANKL, and the decoy receptor osteoprotegerin (OPG), play predominant roles in regulating the bone microenvironment (Khosla 2001). RANKL, which is highly expressed in OBs and osteocytes within bone tissue and stimulates OC activity by binding to its receptor RANK, which is expressed in OCs. OPG serves as a natural inhibitor by binding to RANKL, preventing it from interacting with RANK, and thus reducing bone resorption. The balance between RANKL and OPG within the bone remodelling process is crucial for maintaining bone mass and strength. During bone metastasis, cancer cells induce excessive OC activity and disrupt the balance between OC and OB activities (Roodman 2004). Therefore, denosumab, a human anti‐RANKL monoclonal antibody, is a potent inhibitor of OC bone resorption and a major treatment and prevention strategy for bone metastasis in clinical practice.

PCa bone metastases are uniquely bone‐forming, namely, the osteogenic phenotype, whereas bone metastases of breast cancer and lung cancer are bone‐degrading, namely, the osteolytic phenotype. However, bone resorption markers are also increased in metastatic PCa (Maeda et al. 1997). Moreover, our group previously reported that bone metastatic lesions of PCa showed a tendency to change from an osteolytic to an osteogenic pattern, and relapse was often accompanied by an increase in the number of osteolytic lesions in the clinical course (Shimazaki et al. 1992). These findings highlight the significance of OC activity even in osteogenic PCa bone metastases. However, denosumab does not prolong the overall survival of patients with advanced‐stage PCa (Smith et al. 2012). This inconsistency indicates the existence of underlying mechanisms of pathological OC activation independent of RANKL in the presence of PCa and unknown molecular mechanisms in the process of PCa bone metastasis. Therefore, a more detailed understanding of the interactions between tumour and bone‐resident cells is required to develop novel therapies for advanced‐stage PCa.

Extracellular vesicles (EVs) are small particles with lipid bilayer membranes that are produced by all cell types (Yáñez‐Mó et al. 2015). EVs are widely known as key mediators that shuttle biologically active molecules such as nucleic acids and proteins and contribute to intercellular communication (Raposo and Stoorvogel 2013). Numerous studies have shown that EVs are involved in various biological phenomena, including bone remodelling and cancer progression (Li et al. 2016; Sun et al. 2016; Webber et al. 2010; Peinado et al. 2012; Ono et al. 2014; Tominaga et al. 2015; Yokoi et al. 2017). Indeed, EVs from metastatic PCa cells have been reported to regulate the activity of bone‐resident cells (Tamura et al. 2023). However, almost all previous studies have focused on the role of EVs in PCa cells. Few studies have investigated the role of EVs derived from bone‐resident cells in bone metastasis. Given the importance of pathological OC activation in the process of PCa bone metastasis and the lack of insight into the role of EVs from bone‐resident cells in the presence of PCa, we speculated that EVs from pathological OCs are missing for understanding the molecular mechanism of PCa bone metastasis.

In the present study, we found that pathological OC‐derived EVs play a critical role in disrupting bone homeostasis in metastatic PCa. EVs from OCs corrupted by PCa cells induce OC activation and concurrent OB suppression, driving further bone destruction and tumour progression. PARP1 in OCs and HOXA1 in OBs, which are involved in inflammatory bone destruction, are regulated by pathogenic EV contents in OCs. These molecular responses in bone‐resident cells mediated by pathological OC‐derived EVs are the underlying molecular mechanisms of pathological bone destruction in PCa metastasis.

## 2 Materials and Methods

### 2.1 Cell Cultures

We used two human bone metastatic PCa cell lines: PC‐3M‐luc‐C6 (PC3M) (Xenogen, Alameda, CA, USA), which induces osteolytic bone metastasis, and C4‐2B (American Type Culture Collection [ATCC], Manassas, VA, USA), which induces osteogenic bone metastasis. PC3M, C4‐2B, and the immortalized normal human prostatic epithelial cell line PNT2 (DS Pharma Biomedical Co., Ltd, Osaka, Japan) were cultured in RPMI 1640 medium (Gibco, Thermo Fisher Scientific, Waltham, MA, USA) supplemented with 10%‐inactivated foetal bovine serum (FBS, Gibco, Thermo Fisher Scientific) and 1% antibiotic‐antimycotic (AA) solution (Gibco, Thermo Fisher Scientific) at 37 °C in atmosphere of 95% air and 5% CO_2_. The murine monocytic cell line RAW 264.7 (ATCC), murine pre‐OB cell line MC3T3‐E1 subclone 4 (MC3T3‐E1, ATCC) were cultured in α‐MEM (Sigma–Aldrich, St. Louis, MO, USA) with 10% FBS (Gibco, Thermo Fisher Scientific), 1% AA (Gibco, Thermo Fisher Scientific), and 2 mM L‐Glutamine (Gibco, Thermo Fisher Scientific) at 37°C in atmosphere of 95% air and 5% CO_2_. RAW 264.7 cells were used at passages 4–10. These cell lines were checked for mycoplasma‐free status using the MycoAlert Mycoplasma Detection Kit (Lonza, Basel, Switzerland).

For transwell co‐cultures, 1 × 10^4^ of PC3M or C4‐2B cell lines were seeded into the top of transwell membrane (0.4 µm pore size, Sarstedt, Nümbrecht, Germany) with RAW 264.7 cells (6 × 10^4^ cells/well) or MC3T3‐E1 cells (1 × 10^5^ cells/well) growing in the lower compartment in 6‐well plate in α‐MEM (Sigma–Aldrich) with 10% FBS (Gibco, Thermo Fisher Scientific), 1% AA (Gibco, Thermo Fisher Scientific) and 2 mM L‐Glutamine (Gibco, Thermo Fisher Scientific) in the presence or absence of 50 ng/mL recombinant human soluble RANKL (sRANKL, Oriental Yeast Co., Tokyo, Japan) for RAW 264.7 cells or 50 µg/mL ascorbic acid (FUJIFILM Wako Pure Chemical Corporation, Osaka, Japan) and 10 mM β‐glycerophosphate (FUJIFILM Wako Pure Chemical Corporation) for MC3T3‐E1 cells for 3 days. After removing the transwell inserts, the medium was replaced with a suitable medium for subsequent experiments.

### 2.2 Osteoclastogenesis Assay and TRAP Staining

RAW 264.7 cells were seeded in 24‐well plates at a density of 1.0 × 10^4^ cells/well with α‐MEM (Sigma–Aldrich) containing 10% FBS (Gibco, Thermo Fisher Scientific), 1% AA (Gibco, Thermo Fisher Scientific), and 2 mM L‐Glutamine (Gibco, Thermo Fisher Scientific). After an initial attachment period of 24 h, the following treatments were performed. miR‐mimc and siRNA transient transfection were performed as described bellows, then the medium was changed to α‐MEM (Sigma–Aldrich) containing 10% FBS (Gibco, Thermo Fisher Scientific), 1% AA solution (Gibco, Thermo Fisher Scientific), and 2 mM L‐Glutamine (Gibco, Thermo Fisher Scientific) with or without sRANKL (Oriental Yeast Co.) at the concentration of 10 ng/mL. For EV supplementation treatment, after an initial attachment period of 24 h, the cells were cultured with in combination with 10 µg/mL of purified EVs for 5 days with a change in culture medium every 2–3 days. On Day 6, cells were collected for RNA extraction, fixed with 4% formaldehyde (FUJIFILM Wako Pure Chemical Corporation), and stained with tartrate‐resistant acid phosphatase (TRAP) using a TRAP Staining Kit (Cosmo Bio. Co., Ltd., Tokyo, Japan). Cells were photographed using a BZ‐X710 microscope (Keyence, Osaka, Japan) with a 4× objective (Keyence). For imaging of the entire well, the tiled image stitching function in the BZ‐X Analyzer software (Keyence) was used for image stitching. Mature OC were counted in nine randomly selected fields. TRAP‐positive multinuclear cells containing three or more nuclei were considered mature OCs. The timeline of cell culture is outlined in Figure S1.

The effects of the inhibitory agents on osteoclastogenesis were evaluated. After transwell co‐culture as described above, the medium was replaced to α‐MEM (Sigma–Aldrich) containing 10% FBS (Gibco, Thermo Fisher Scientific), 1% AA (Gibco, Thermo Fisher Scientific), 2 mM L‐Glutamine (Gibco, Thermo Fisher Scientific), 50 ng/mL sRANKL (Oriental Yeast Co.). 10 µg/mL of denosumab (Ranmark; Daiichi‐Sankyo Co. Ltd., Tokyo, Japan) was supplemented after additional 24 h incubation, and then the cells were observed and photographed by the BZ‐X710 microscope (Keyence) with TRAP staining 24 h after denosumab supplementation. The optimum concentration of denosumab was evaluated by the application of different concentrations (10–1000 µg/mL) of denosumab to the co‐cultured cells.

### 2.3 Osteoblastogenesis Assay and ALP and Alizarin Red Staining

MC3T3‐E1 cells were seeded in 24‐well plates at a density of 4.0 × 10^4^ cells/well for miR‐mimc and siRNA transient transfection experiments or in 96‐well plates at a density of 1.0×10^4^ cells/well for EV supplementation experiments with α‐MEM (Sigma–Aldrich) with 10% FBS (Gibco, Thermo Fisher Scientific), 1% AA (Gibco, Thermo Fisher Scientific) and 2 mM L‐Glutamine (Gibco, Thermo Fisher Scientific). After an initial attachment period of 24 h, the following treatments were performed. miR‐mimc and siRNA transient transfection were performed as described bellows, then the medium was changed to α‐MEM (Sigma–Aldrich) containing 10% FBS (Gibco, Thermo Fisher Scientific), 1% AA solution (Gibco, Thermo Fisher Scientific) and 2 mM L‐Glutamine (Gibco, Thermo Fisher Scientific) with or without 50 µg/mL ascorbic acid (FUJIFILM Wako Pure Chemical Corporation) and 10 mM β‐glycerophosphate (FUJIFILM Wako Pure Chemical Corporation) with a change in culture medium every 2–3 days. For EV supplementation treatment, after an initial attachment period of 24 h, the cells were cultured with in combination with 10 µg/mL of purified EVs for each period of time with a change in culture medium every 3 days. Cells were collected for RNA extraction at the indicated time points, fixed with 4% formaldehyde (FUJIFILM Wako Pure Chemical Corporation), and stained for alkaline phosphatase (ALP) using an ALP staining kit (Cosmo Bio. Co., Ltd.) on Day 10. In addition to ALP staining, calcium deposition was examined using an Alizarin Red staining kit (Cosmo Bio. Co.) on Day 21. The timeline of cell culture is outlined in Figure S1.

Cells were photographed using a BZ‐X710 microscope (Keyence). For imaging of the entire well, a BZ‐X710 microscope (Keyence) with a 4x objective was used to capture tiled images, and the image stitching function in the BZ‐X Analyzer software (Keyence) was used for image stitching.

### 2.4 Cell Proliferation Assay

Cell viability was measured using a Cell Counting Kit‐8 (CCK‐8; Dojindo, Kumamoto, Japan) according to the manufacturer's instructions. After an initial attachment period of 24 h in 96‐well plates, the cells were treated with EVs or miR‐mimics and transiently transfected with siRNA, as described previously. 48 or 96 h later, 10 µL of CCK‐8 reagent was added to the cell medium and incubated at 37°C for 4 h. The absorbance was measured at 450 nm using a Synergy H4 Microplate Reader (BioTek, Winooski, VT, USA).

### 2.5 miR‐Mimic and siRNA Transient Transfection

The cells were transfected with Lipofectamine RNAiMAX Reagent (Thermo Fisher Scientific) according to the manufacturer's protocol. RAW 264.7 cell suspension of 1.0 × 10^4^ cells/well MC3T3‐E1 cell suspension of 4.0 × 10^4^ cells/well (in α‐MEM (Sigma–Aldrich) containing 10% FBS without antibiotics) was seeded in 24‐well plates and incubated for 24 h. For transfection of the miR‐mimics, mmu‐miR‐1963, mmu‐miR‐5112 and miR‐mimic NC (Dharmacon, Larayette, CO, USA) were used for each transfection at a final concentration of 20 nM. For the transfection of siRNA, siRNA‐Parp1, siRNA‐Hoxa1 and siRNA NC (Dharmacon) were used for each transfection at a final concentration of 20 nM. After 6 h of incubation, the medium was changed to α‐MEM (Sigma–Aldrich) with 10% FBS (Gibco, Thermo Fisher Scientific), 1% AA (Gibco, Thermo Fisher Scientific) and 2 mM L‐Glutamine (Gibco, Thermo Fisher Scientific) in the presence or absence of recombinant mouse RANKL (GST‐RANKL, Oriental Yeast Co., Tokyo, Japan) at a concentration of 10 ng/mL for RAW 264.7 cells or ascorbic acid (FUJIFILM Wako Pure Chemical Corporation) at a concentration of 10 ng/mL for MC3T3‐E1subclone 4 cells. *Parp1* and osteoclastic marker gene (Trap and Nfatc1) expression in RAW 264.7 cells or *Hoxa1*, and osteoblastic marker gene (Col1a1, Alpl and Bglap) expression in MC3T3‐E1 cells were examined by qRT‐PCR as described above. TRAP and ALP staining were performed as previously described. The miR‐mimic and siRNA products used in this study are listed in Table S1.

### 2.6 In Vivo Studies

NOD SCID (NOD.CB17‐Prkdc^scid^/J) mice (Charles River Laboratories Japan, Inc., Kanagawa, Japan) were used for the experiments. Animal experiments were performed in compliance with the guidelines of the Institute for Laboratory Animal Research, National Cancer Center Research Institute (Number: T19‐003‐M02), and considering the 3Rs stipulated by the Animal Welfare Management Act. The caudal artery injection procedure in mice has been previously reported (Kuchimaru et al. 2018). Luciferase‐expressing PC3M (5.0 × 10^5^ cells/100 µL diluted in phosphate‐buffered saline [PBS]) was injected into the caudal artery of 5‐week‐old male SCID mice. Following treatment, the tumours were monitored using an IVIS Lumina II (PerkinElmer, Waltham, MA, USA) every week until bone metastatic tumours appeared in the femur or tibia. After the establishment of bone metastases in almost all the mice, we divided them into two groups: a miRNA control injection group and a miR‐mimic injection group.

For the in vivo miRNA delivery experiments, mice were treated via caudal artery injection with 40 µg of NC or 20 µg of mmu‐miR‐1963 and 20 µg of mmu‐miR‐5112 mimic mix‐packaged with in vivo‐jetPEI (Polyplus Transfection, Illkirch, France) in a total volume of 100 mL/mouse, prepared according to manufacturer recommendations.

After each monitoring period, the mice were evaluated using a micro‐computed tomography (micro‐CT) system, R‐mCT2 (Rigaku Co., Tokyo, Japan), on the day of sacrifice. To collect the tissues, both legs were separated from the pelvis and fixed in 4% paraformaldehyde in PBS. After tissue collection, tissue embedding was performed at the Koto Microbiology Laboratory (Tokyo, Japan) and haematoxylin and eosin (HE) staining was performed.

### 2.7 TRAP and ALP Double Staining

TRAP and ALP double staining of tissues was performed on thin sections using the TRAP/ALP Stain Kit (FUJIFILM Wako Pure Chemical Corporation) according to the manufacturer's instructions. Briefly, paraffin sections were fully dewaxed, and TRAP and ALP were added sequentially. TRAP and ALP were incubated in a moist chamber at room temperature (RT) for 30 min. The sections were dried and photographed under a BZ‐X710 microscope (Keyence).

### 2.8 Immunohistochemistry

The paraffin sections were fully dewaxed. Next, antigen unmasking was conducted by heating the samples in Immunosaver (NEM, Tokyo, Japan) for 3 min at 120 °C. After endogenous peroxidase inactivation, permeabilization, and blocking using Protein Block Serum‐Free (Dako, Glostrup, Denmark), the sections were incubated overnight at 4°C with primary antibodies against the antigens: anti‐LAT1 (#5347, CST, Danvers, MA, USA) diluted in REAL antibody diluent (Dako). Next, the sections were incubated for 1 h at RT with ImPRESS IgG‐369 peroxidase kits (Vector Laboratories, Newark, CA, USA), and color development was performed using the ImmPACT DAB substrate kit (Vector Laboratories) under a light microscope, followed by counterstaining with Mayer's hematoxylin (Applied Biosystems, Waltham, MA, USA) for 2 min. The sections were dried, and the cells were photographed using a BZ‐X710 microscope (Keyence).

### 2.9 Preparation of Conditioned Medium and EV Isolation

The cells were washed with PBS, and the culture medium was replaced with Advanced DMEM (Gibco, Thermo Fisher Scientific) for RAW 264.7 cells, containing an AA mix solution (Gibco, Thermo Fisher Scientific), 2 mM L‐Glutamine (Gibco, Thermo Fisher Scientific) and 50 ng/mL sRANKL (Oriental Yeast Co.). For PC3M and C4‐2B cells, the culture medium was changed to Advanced RPMI 1640 medium (Gibco, Thermo Fisher Scientific) containing an AA mix solution (Gibco, Thermo Fisher Scientific) and 2 mM L‐Glutamine. After incubation for 48 h, the conditioned medium was collected, and EVs were isolated using a differential ultracentrifugation protocol, as we previously reported (Yoshioka et al. 2014). Briefly, the conditioned medium was centrifuged at 2000 × *g* for 10 min at 4°C to remove contaminating cells. The resulting supernatants were then transferred to fresh tubes and filtered through a 0.22 µm filter (Millipore, Billerica, MA, USA). The filtered conditioned medium was centrifuged for 70 min at 210,000 ×*g* (35,000 rpm) using an SW41Ti rotor (Beckman Coulter, Rea, CA) at 4°C to pellet the enriched EVs. The pellets were washed with 11 mL of PBS (−) and ultracentrifuged at 210,000 × *g* (35,000 rpm) for 70 min using an SW41Ti rotor. The EV pellets were stored in a refrigerator at 4°C until use. The protein concentration of the putative EV fraction was determined using a Quant‐iT Protein Assay with a Qubit 2.0 Fluorometer (Thermo Fisher Scientific). To determine the size distribution of the EVs, nanoparticle tracking analysis was conducted using the Nanosight LM10‐HS system (Malvern Panalytical, Malvern, UK) on samples diluted 400‐fold with PBS (−) for analysis. All procedures were designed in accordance with the MISEV2023 guidelines (Welsh et al. 2024).

### 2.10 PKH26‐Labelled EV Uptake Assay

Purified EVs derived from OC cells were labelled using the PKH26 Red Fluorescent Labelling Kit (Sigma‒Aldrich) according to the manufacturer's instructions. Briefly, EVs were incubated with 2 µM PKH26 for 5 min, washed four times with PBS through a 100‐kDa filter (Microcon YM‐100, Millipore) to remove excess dye. MC3T3‐E1 cells seeded on a 35 mm glass‐bottom dish (1 × 10⁵ cells/dish) were supplemented with PKH26‐labelled EVs (2 × 10⁹ particles/dish). After 17 h incubation in the presence of ascorbic acid (50 µg/mL) at 37°C, images were acquired using a LSM700 confocal microscope (Carl Zeiss, Oberkochen, Germany).

### 2.11 Immunoblotting

Whole‐cell lysates were prepared using the Mammalian Protein Extract Reagent (M‐PER; Thermo Scientific). After removing the culture medium, cells in a 24‐well culture plate were washed with PBS and 300–500 µL M‐PER was then added. Whole‐cell lysates were transferred to 1.5 mL tube and sonicated. Proteins (cell lysate, EVs) were load onto Mini‐PROTEAN TGX Gel (4%–15%, Bio‐Rad, Hercules, CA, USA) and electro transferred onto a PVDF membrane using Trans‐Blot Turbo Transfer System (Bio‐Rad). After blocking with Blocking One (Nacalai Tesque, Kyoto, Japan), membranes were incubated for 1 h at room temperature with primary antibodies against the following antigens: anti‐GM‐130 ([EP892Y], ab52649, Abcam, dilution 1:1000), anti‐actin (clone C4, MAB1501, Millipore, dilution 1:1000), anti‐CD9 ([EM‐04], ab82390, Abcam, dilution 1:1000), anti‐CD63 (D263‐3, MBL, dilution 1:1000). Secondary antibodies (horseradish peroxidase‐conjugated anti‐mouse IgG, NA931, or horseradish peroxidase‐conjugated anti‐rabbit IgG, NA934; GE Healthcare) were used at a dilution of 1:5000. The membranes were then incubated with ImmunoStar LD reagent (Wako, Osaka, Japan).

### 2.12 RNA Extraction and PCR Analysis

Total RNA and miRNA were extracted from cultured cells and purified EVs (10 µg) using QIAzol and the miRNeasy Mini Kit (Qiagen, Hilden, Germany) according to the manufacturer's protocols. Total RNA was converted to first‐strand complementary DNA (cDNA) using a High‐Capacity cDNA Reverse Transcription Kit (Applied Biosystems). Real‐time PCR was performed in triplicate or quadruplicate with cDNA using Platinum SYBR Green qPCR SuperMix UDG (Thermo Fisher Scientific). Data were collected and analysed using StepOne Real‐Time PCR System and StepOne Software v2.3 (Applied Biosystems). All mRNA quantification data from cultured cells were normalized to the expression of β‐actin and All primer sequences are shown in Table S2.

### 2.13 Next‐Generation Sequencing (NGS)

Total RNA and EV‐associated miRNAs were isolated from cultured cells and EVs were purified EVs by QIAzol and the miRNeasy Mini Kit (Qiagen, Hilden, Germany) according to the manufacturer's protocols, as described above. Prior to the NGS analysis, the quality of the RNA samples was evaluated using an Agilent 2100 Bioanalyzer (Agilent Technologies, Santa Clara, CA, USA) with an Agilent RNA 6000 pico kit and Agilent small RNA kit (Agilent Technologies).

Total RNA of the eight samples of OCs were sent to DNA Chip Research Inc. (Tokyo, Japan), where RNA library preparation and sequencing were performed. RNA libraries were prepared using the NEBNext Ultra II Directional RNA Library Prep for Illumina, NEBNext rRNA Depletion Kit, and NEBNext Multiplex Oligos for Illumina (96 Unique Dual Index Primer Pairs), and the yields were evaluated using an Agilent 2100 BioAnalyzer. RNA sequencing was performed using the Illumina NextSeq 500 system (Illumina, San Diego, CA, USA).

EV‐associated miRNAs from the seven samples were sent to DNA Chip Research, Inc. (Tokyo, Japan) for small‐RNA library preparation and sequencing. Small RNA libraries were prepared using the QIAseq miRNA library Kit and QIAseq miRNA NGS 96 Index IL (Illumina), and the yields were evaluated using the Agilent 2100 BioAnalyzer. Small RNA sequencing was performed using an Illumina NextSeq 500 system.

Total RNA from the six samples used for detecting miRNA target genes was sent to Azenta Japan Co. (Tokyo, Japan) for RNA library preparation and sequencing.

RNA libraries were prepared using the NEBNext Ultra II Directional RNA Library Prep for Illumina, NEBNext rRNA Depletion Kit, and NEBNext Poly(A) mRNA Magnetic Isolation Module (NEB). Yields were evaluated using the Qubit DNA Assay (Thermo Fisher Scientific) and TapeStation D1000 ScreenTape (Agilent Technologies). RNA sequencing was performed using a NovaSeq 6000 (Illumina).

### 2.14 Luciferase Reporter Assay

The 3' UTR of *Parp1* and *Hoxa1*, containing the target sequences (*Parp1*: positions 426–432 and 500–506, *Hoxa1*: positions 502–509) as predicted by TargetScanMouse, was cloned from total RNA of RAW 264.7 and MC3T3‐E1 cells using PCR. The following primers were used for cloning: *Parp1*, Forward: 5'‐CAGCTAGCTTGACAGGTTAAAGGGCTCTGG‐3' including Nhe I restriction site, Reverse: 5'‐CAGTCGACTAGTACTGATTACAGACGGTTC‐3' including Sal I restriction site. *Hoxa1*, Forward: 5'‐CAGCTAGCTTCCTGGGCTGGGATTTCTTAC‐3' including Nhe I restriction site, Reverse: 5'‐CAGTCGACTAGCCCATAATCACACATGAAC‐3' including Sal I restriction site. 3'polyadenylate overhangs were added to the PCR products after treatment with Taq polymerase (72°C, 15 min). The plasmid was cloned into the pGEM‐T easy vector (A137A; Promega, Madison, WI, USA). The amplified products were ligated into the Nhe I and Sal I sites of the 3’UTR of the firefly luciferase gene in the pmirGLO Dual‐Luciferase miRNA Target Expression vector (E1330, Promega) to generate pmiR‐Parp1 and pmiR‐Hoxa1. Site‐directed mutagenesis was performed on the seed sequences of *Parp1* (426‐432), *Parp1* (500‐506) and *Hoxa1* using PrimeStar Max DNA Polymerase (R045, Takara, Japan) for PCR amplification. The following primers were used for site‐directed mutagenesis: *Parp1* (426‐432), Forward: 5'‐ATTTGGCGATCAAAATAAAAGTTAATTTC‐3' Reverse: 5'‐TTTGATCGCCAAATAAATGCTTAACATT‐3. ’ *Parp1* (500‐506), Forward: 5'‐TCTGATTATCGTTTGTTTTGCTTGGTTTT‐3' Reverse: 5'‐AAACGATAATCAGACTAAGAGGGGGACA‐3.’ *Hoxa1*, Forward: 5'‐TTTCGCTTACAATCTGGGGAGCTCCTGGCC‐3' Reverse: 5'‐AGATTGTAAGCGAAAGGAAGAGAGAGTCT‐3.’

The HEK293 cells were cultured in 96‐well plates at a density of 1 × 10^4^ cells/well. After 24 h of initial cell attachment, each construct was co‐transfected with mmu‐miR‐5112 and mmu‐miR‐1963 or a NC mimic using the DharmaFECT Duo Transfection Reagent (Dharmacon, Horizon Discovery). The cells were harvested 48 h after transfection and *Rennila* luciferase activity was measured and normalized to firefly luciferase activity using a Dual‐Glo Luciferase Assay System (Promega). Relative luminescence was calculated by normalizing the values against the transfection of the NC mimic group. All assays were repeated at least thrice, and representative results are shown.

### 2.15 ELISA

Quantitation of secreted IL‐1β in the conditioned medium were measured by ELISA using the mouse Interleukin one beta detection ELISA kit (#6705; Chondrex, Inc, Woodinville, WA, USA) according to the manufacturer's instructions. The absorbance was measured using a Synergy H4 Microplate Reader (BioTek).

### 2.16 Statistical Analysis

All data are presented as means ± standard deviation (SD). Comparisons between two groups were performed using Student's *t*‐test. One‐way analysis of variance (ANOVA) was used to determine significant differences among the three groups, followed by Tukey's or Dunnett's multiple comparison test. Statistical *p* value < 0.05 was considered as statistically significant. The statistical software used was PRISM version 8 (GraphPad Software Inc., CA, USA).

## 3 Results

### 3.1 Induction of Pathological OCs by Metastatic PCa Cells

**FIGURE 1:**
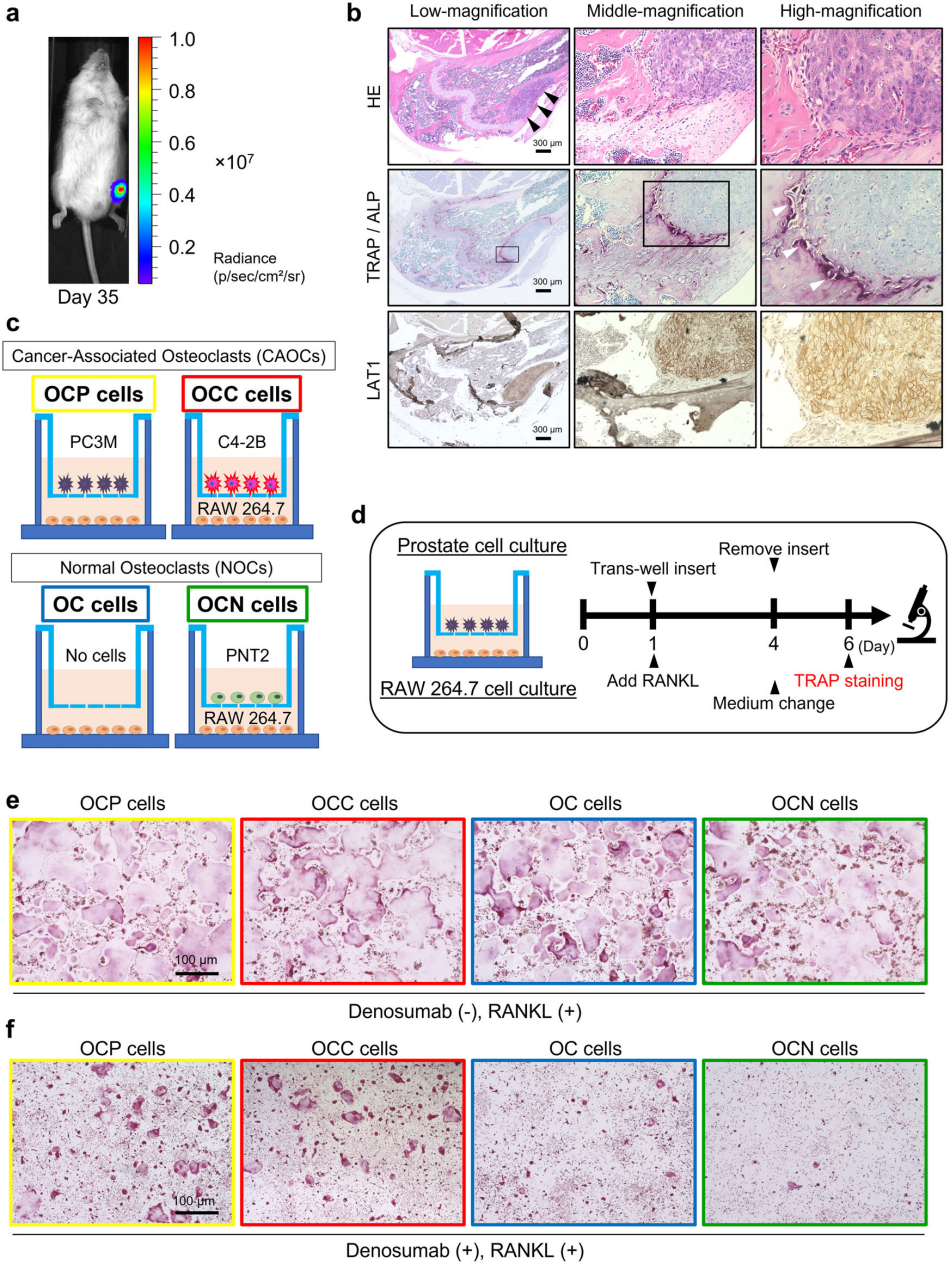
**Metastatic PCa cells promote OC activity and induce pathological phenotype. (a)** Representative IVIS image showing PCa‐transplanted xenograft mice (*N* = 3) on Day 35. Tumours in the hind limbs were evaluated based on the photon radiance of cancer cell bioluminescence. **(b)** Representative images of HE‐, osteoclastic and osteoblastic marker TRAP/ALP‐ and cancer‐specific marker LAT1 stained sections of bone metastatic sites in the xenografted mouse which was shown in (a). All images were obtained with an all‐in‐one fluorescence microscope using a 4x objective for low‐, 10x objective for middle‐, and 40x objective for high‐magnification. Black arrowheads indicate the tumour metastatic site, and white arrowheads indicate OCs. The metastatic tumour developed in the femur. Scale bar indicates 300 µm. **(c)** Schematic images of the transwell co‐culture system for obtaining cancer‐associated OCs (CAOCs) and normal osteoclasts (NOCs). OC precursor RAW 264.7 cells were grown into mature OCs in the lower compartment. Mature OCs with osteolytic PC3M cells (OCP cells) and mature OCs with osteoblastic C4‐2B cells (OCC cells) were used as CAOCs. Mature OCs with blank transwells (OC cells) and mature OCs with PNT2 (OCN cells) were prepared as control NOCs. **(d)** Schematic images of transwell co‐culture for observing CAOCs and NOCs. **(e)** Representative TRAP staining images of each OC. Bars represent 100 µm. (**f)** Representative TRAP staining images of each OCs treated with denosumab at the concentration of 10 µg/mL. Bars represent 100 µm.

First, we sought to confirm whether OC‐induced bone resorption is a necessary priming event for tumour progression after cancer cells have colonized the bone. The human PCa cell line PC3M, which can easily establish tumour metastases showing an osteolytic reaction, was implanted via caudal artery injection to obtain a mouse bone metastasis specimen. PC3M cells stably expressed luciferase; thus, metastatic processes were monitored noninvasively using an in vivo imaging system (IVIS) every week. We observed intense luciferase expression in the hind limbs of the mice 5 weeks after transplantation using IVIS. Histopathological analysis revealed that an increased presence of bone‐resorbing tartrate‐resistant acid phosphatase (TRAP)‐positive cells in the peripheral area of the tumour metastasis compared to the control (Figures 1a,b and S2a–c). Previous in vivo studies have shown that PCa cells entering the bone act as triggers and disrupt bone resorption, followed by the deposition of new pathological bone tissue (Yonou et al. 2004). Our observations were consistent with these studies and prompted us to focus on bone‐resorbing OCs initiating bone tumour metastasis.

Next, we cultured the murine monocytic cell line RAW 264.7, which has the potential to differentiate into OC‐like cells, with the metastatic PCa cell lines to investigate the relationship between PCa cells and OCs. It is well known that PCa bone metastases uniquely show osteogenic phenotype. Therefore, we utilized C4‐2B cells, which show an osteogenic reaction, in addition to PC3M cells, which show an osteolytic reaction, to investigate the molecular mechanisms of both types of PCa. We prepared four distinct OC types using transwell co‐culture system: OCs differentiated from RAW 264.7 cells (OC cells) with blank inserts, OCs co‐cultured with normal prostate epithelial cells (OCN cells), osteolytic PCa (PC3M) cells (OCP cells), and osteoblastic PCa (C4‐2B) cells (OCC cells) (Figure 1c,d). Hereinafter, we refer to OCs co‐cultured with PCa cells, namely, OCP and OCC cells, as cancer‐associated OCs (CAOCs), and OCs co‐cultured with no cells or normal epithelial cells as normal OCs (NOCs). As shown in Figure 1e, mature OCs, which are TRAP positive fused polykaryons arising from multiple individual cells (Boyle et al. 2003), were observed 120 h after transwell insertion and addition of RANKL. The four types of OCs appeared to have similar cellular morphology, and the expression levels of TRAP appeared to be almost the same under these conditions (Figures 1e and S3a–e and Table S3). Surprisingly, treatment with denosumab, an anti‐RANKL antibody agent widely used in the clinic, revealed differences in characteristics between CAOCs and NOCs; OCP and OCC cells demonstrated the cell activity as TRAP positive fused polykaryons even in the presence of denosumab, compared to OC and OCN cells (Figures 1f and S3a–f and Table S3). This finding suggests the existence of RANKL‐independent molecular mechanisms underlying pathological OC activation.

### 3.2 Characteristics of Pathological OCs Corrupted by PCa Cells

**FIGURE 2:**
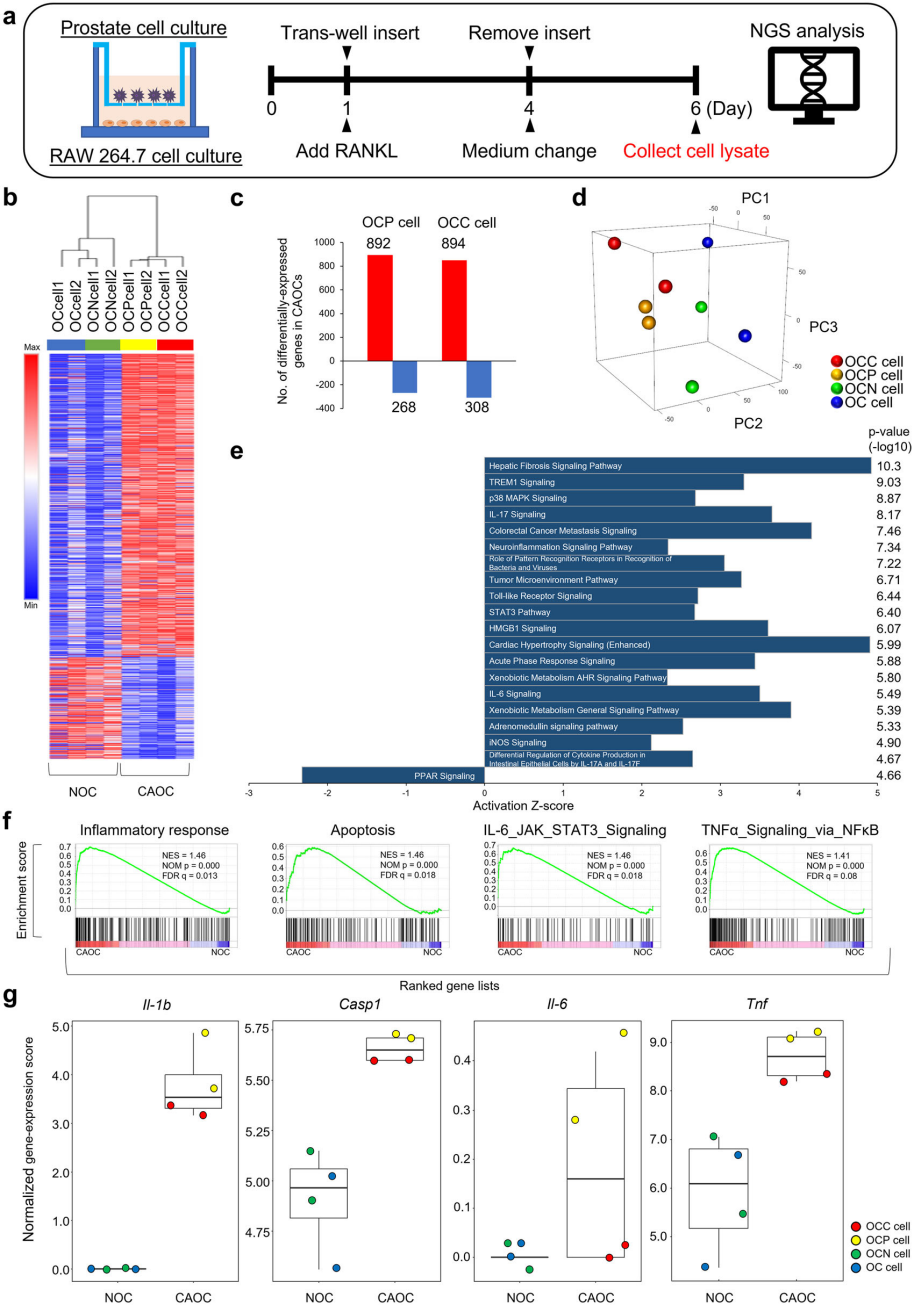
**Inflammatory‐related signalling pathways are enhanced in CAOCs. (a)** Schematic protocol of co‐culture and gene expression analysis of CAOCs. **(b)** Heat map showing differentially expressed genes (CAOC vs. OCN, change >2.0‐fold and *p *< 0.05) in RAW 264.7, co‐cultured with PCa cells (*n* = 2) in the presence of RANKL. **(c)** The number of differentially expressed genes in mature OCs co‐cultured with PC3M cells (OCP cells) or C4‐2B cells (OCC cells) compared to that in OCN cells co‐cultured with PNT2 cells (OCN cells). **(d)** PCA of gene expression of each type of OCs. **(e)** Pathway analysis of selected genes that were significantly upregulated in CAOCs compared to OCN cells. **(f)** GSEA of CAOC versus NOC, highlighting pro‐inflammatory phenotypes. NES: normalized enrichment score. *p* value was calculated using GSEA. **(g)** Expression levels of *Il‐1b*, *Casp1*, *Il‐6* and *Tnf* in gene sets of inflammation‐related genes. OC co‐cultured with a blank insert (OC cells) or PNT2 (OCN cells), and OC co‐cultured with PC3M (OCP cells) or C4‐2B (OCC cells) are presented. The blue, green, yellow and red dots represent the OC, OCN, OCP and OCC cell data, respectively.

To understand the molecular characteristics of CAOCs, we performed next‐generation sequencing (NGS) analysis of CAOCs and NOCs (Figure 2a,b). We found 892 upregulated genes (>2‐fold, *p *< 0.05) and 268 downregulated genes (<0.5‐fold, *p *< 0.05) in OCP cells compared to those in OCN cells. Similarly, we found 894 upregulated genes (>2‐fold, *p *< 0.05) and 308 downregulated genes (<0.5‐fold, *p *< 0.05) in OCC cells (Figure 2c). Principal component analysis (PCA) mapping of NGS data showed convergence between OCP and OCC cells (Figure 2d). In contrast, PCA concurrently showed divergence between CAOCs (OCP and OCC cells) and NOCs (OC and OCN cells), reflecting the impact of co‐culturing with PCa cells (Figure 2d). These findings indicate that PCa cell lines distinctly affect OC phenotypes in the co‐culture system. To further investigate these CAOC phenotypes, QIAGEN Ingenuity Pathway Analysis was performed (Figure 2e). Pathway analysis revealed that significant changes in several inflammatory signalling pathways, including interleukin families such as IL‐6 and IL‐17 signalling, were induced by co‐culture with PCa cells. Consistent with this analysis, gene set enrichment analysis (GESA) also showed significant enrichment of gene sets for inflammatory‐related signalling pathways including “inflammatory response,” “Apoptosis,” IL‐6_JAK_STAT3 signalling'' and “TNFα signalling via NF‐kB” (Figure 2f and Table S4). In previous studies on arthritis‐associated bone destruction, various inflammatory cytokines and their combinations have been reported to induce pathologically activated OCs, leading to extraordinary osteolysis. In particular, IL‐1β, IL‐6, IL‐17 and TNFα are reported to be deeply associated with RANKL‐independent OC differentiation (Mbalaviele et al. 2017). Among these inflammatory cytokines, *Il‐1b* and *Tnf* were significantly upregulated in OCP and OCC cells, whereas *Il‐6* was increased only in OCP cells in our data set (Figures 2g and S4a). *Casp1* encoding CASP1, which is directly involved in the processing of pro‐IL‐1β and the secretion of IL‐1β, was also significantly upregulated in OCP and OCC cells. As shown in Figure 1e,f, OCC cells, which did not exhibit an increase in *Il‐6* expression, still demonstrated the activity as TRAP positive cells even under anti‐RANKL therapy; thus, IL‐6 is not an essential factor in the underlying RANKL‐independent mechanism. Above all, *Il‐1b* elevation is identified as a quite specific molecular characteristic in CAOC (Figure 2g).

Taken together, these findings indicate that PCa cells induce inflammatory state in OCs, that is, corrupt surrounding OCs and change them into pathological phenotype producing inflammatory cytokines, especially IL‐1β. Moreover, this phenotypic change was associated with the molecular mechanisms of pathological OC activation, independent of RANKL.

### 3.3 EVs From Pathological OCs Show Distinct Functions and microRNA Contents

**FIGURE 3:**
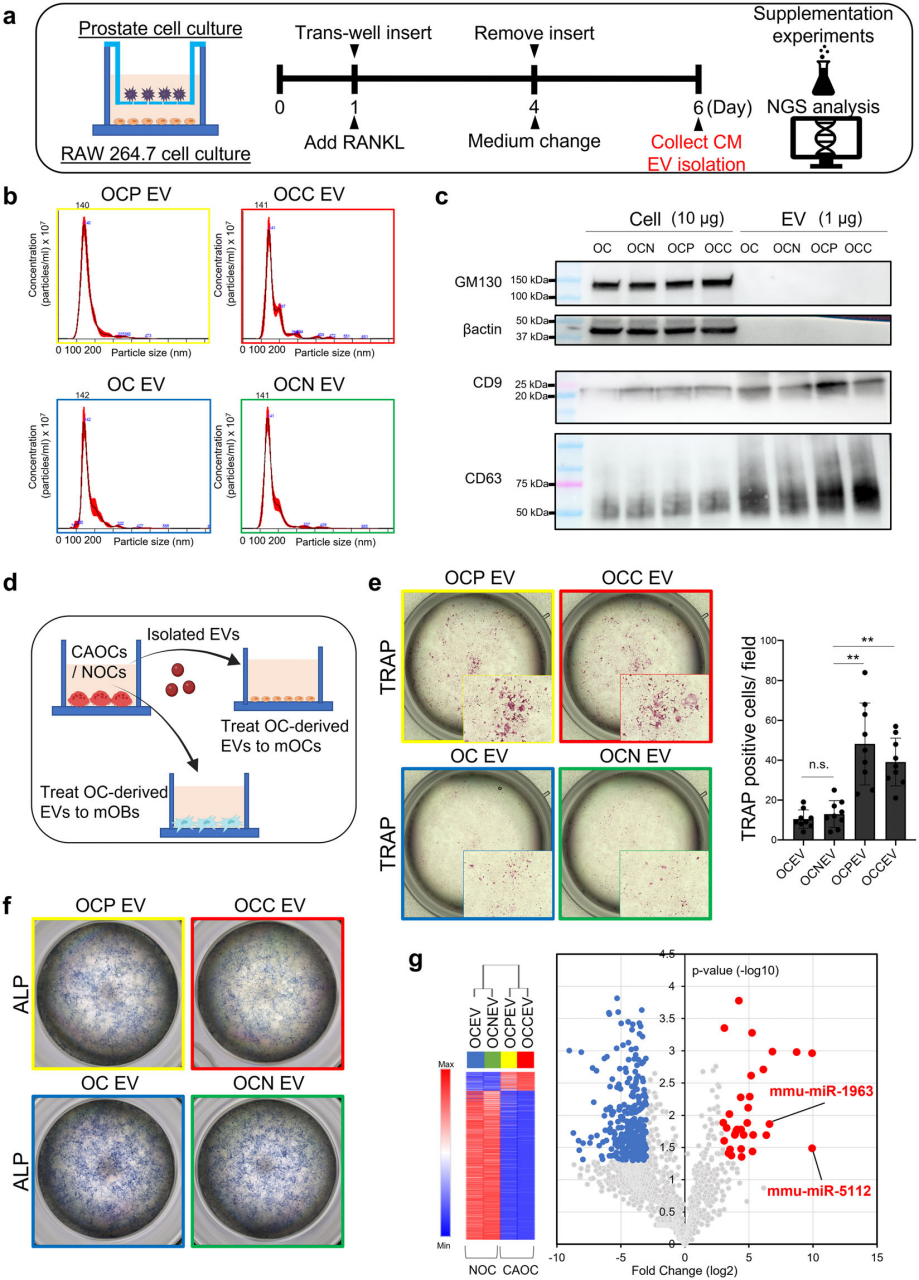
**EVs from CAOCs transferring various miRNAs promote further OC activity and inhibit OB activity. (a)** Schematic protocol for coculture and preparation of conditioned medium (CM) and EVs. **(b)** Nanoparticle tracking analysis showing particle sizes of OC, OCNEVs, OCPEVs and OCC EVs. The vertical axis in the graphs shows the number of EV particles (×10^7^)/mL and the horizontal axis indicates the particle size (nm) of the EVs. **(c)** Immunoblot analysis of conventional EV‐positive and EV‐negative markers. 10 µg/lane for cells and 1 µg/lane for EVs. **(d)** Schematic protocol for the EV supplementation experiments in maturing OCs (mOCs) and mineralizing OBs (mOBs) for each type of OC‐derived EVs. This figure was created using Bio‐Render.com. **(e)** TRAP staining of mOC treated with each OC‐derived EVs (left panel) and TRAP TRAP‐positive cell count (right panel). Error bars represent standard deviation (SD) of the mean (*n* = 9). **p < 0.01, One‐way ANOVA. RAW 264.7 cells were seeded in 24‐well plates in the presence of RANKL (10 ng/mL). The cells were stained for TRAP activity after another 5 days. **(f)** ALP staining of mOB treated with OC‐derived EVs. The MC3T3‐E1 cells were seeded in 96‐well plates in the presence of ascorbic acid and β‐glycerophosphate. The cells were stained for ALP activity after another 9 days. **(g)** Heat map showing differentially expressed miRNAs (CAOC EVs vs. OCN EVs, change>2.0‐fold, and *p*<0.05) enriched in OC, OCN, OCP and OCC EVs (left panel). Volcano plot showing differentially expressed miRNAs in CAOC and NOC EVs. Significant differences (change>3.0‐fold, *p *< 0.05) are indicated in red (enriched CAOC‐EVs) and blue (enriched NOC‐EVs). Representative miRNAs in the CAOC EVs are shown (right panel).

We then investigated how CAOCs, which are pathologically activated OCs, communicate with other bone‐resident cells. Most PCa bone metastases eventually show bone formation, namely, an osteogenic pattern, in the clinical course (Roodman 2004). Alterations from osteolytic to osteogenic patterns were observed in the same lesion, regardless of the effects of endocrine therapy (Shimazaki et al. 1992; Yonou et al. 2004). Accumulating evidence has demonstrated that EVs play key roles in bone homeostasis under physiological and pathological conditions (Tamura et al. 2023). Recent studies have suggested that EVs produced by OCs are important cues for switching from the bone‐resorbing phase to the bone‐forming phase during normal homeostasis (Ikebuchi et al. 2018). Therefore, we hypothesized that EVs from OC corrupted by PCa cells could contribute to elucidating the underlying mechanism of bone metastasis in PCa. To test this hypothesis, we prepared EVs from conditioned medium (CM) of CAOCs and NOCs using ultracentrifugation (Figure 3a). The isolated EVs were characterized using cryo‐transmission electron microscopy (TEM), nanoparticle tracking analysis (NTA) system and western blotting (WB) of conventional EV‐negative and‐positive markers (Figures S5a and 3b,c). Cryo‐TEM analysis revealed the characteristic small EV morphology, displaying a bilipid layered structure with a size of approximately 100–200 nm, consistent with measurements obtained using the NTA system. WB analysis was performed to further validate the EV preparation. The EV‐negative marker GM130 was not detected in EV samples, whereas the EV‐positive markers CD9 and CD63 were detected and enriched in EV samples compared to whole cell lysate protein samples. A correlation was observed between the particle numbers quantified by NTA and the protein concentration of EV samples (Table S5 and Figure S5b), highlighting the reliability of these metrics for EV characterization. To investigate the effects of CAOC EVs, we applied them to normally maturing OC (mOC) and mineralizing OB (mOB) at 10 µg/mL concentration in vitro (Figure 3d). As shown in Figure 3e,f, CAOC EVs promoted mOC activity and inhibited mOB activity. OCP EVs, which are EVs derived from OCs with osteolytic PC3M cells, and OCC EVs, which are EVs derived from OCs with osteoblastic C4‐2B cells, had similar effects on OC and OB differentiation. These results for OC‐derived EVs were in accordance with the results of CM culture of each OCs (Figure S6a). We performed EV uptake tests and confirmed the uptake of these isolated OC‐derived EVs into OBs by PKH26‐labelled EVs before the EV supplementation experiments (Figure S6b). Furthermore, we confirmed the suppressive effect of EVs on OB differentiation at the gene expression level (Figure S6c). These results prompted us to investigate whether EV cargo affects bone homeostasis and tumour progression. Among the EV cargos, microRNAs (miRNAs) have been studied extensively over the past decades and are considered powerful molecules with multiple target genes that are critically involved in the post‐transcriptional regulation of gene expression. Thus, we performed NGS to analyse the small RNA profiles in OCP and OCC EVs compared to those in OC and OCN EVs (Figure 3g). A total of 1391 miRNAs were found in CAOC‐derived EVs. The miRNA profiles of OCP and OCC EVs showed a similar pattern, which was in accordance with the result that OCP and OCC cells showed similar gene expression patterns and that OCP and OCC EVs had a similar impact on OC and OB differentiation. Moreover, only 32 enriched miRNAs were significantly differentially expressed in CAOC EVs compared to NOC EVs (Figure 3g). Among the 32 miRNAs, mmu‐miR‐1963 and mmu‐miR‐5112 were previously reported to be modulators of bone homeostasis or the tumour microenvironment (Wang et al. 2018; Lee et al. 2017). These two miRNAs were more abundant in CAOC EVs than the other miRNAs (Table S6), thus considered to be selectively packaged into CAOC EVs. Therefore, we focused on these miRNAs to elucidate the role of CAOC EVs in bone homeostasis under pathological conditions.

### 3.4 EV‐mmu‐miR‐5112 Promote OC Activity Targeting *Parp1*

**FIGURE 4:**
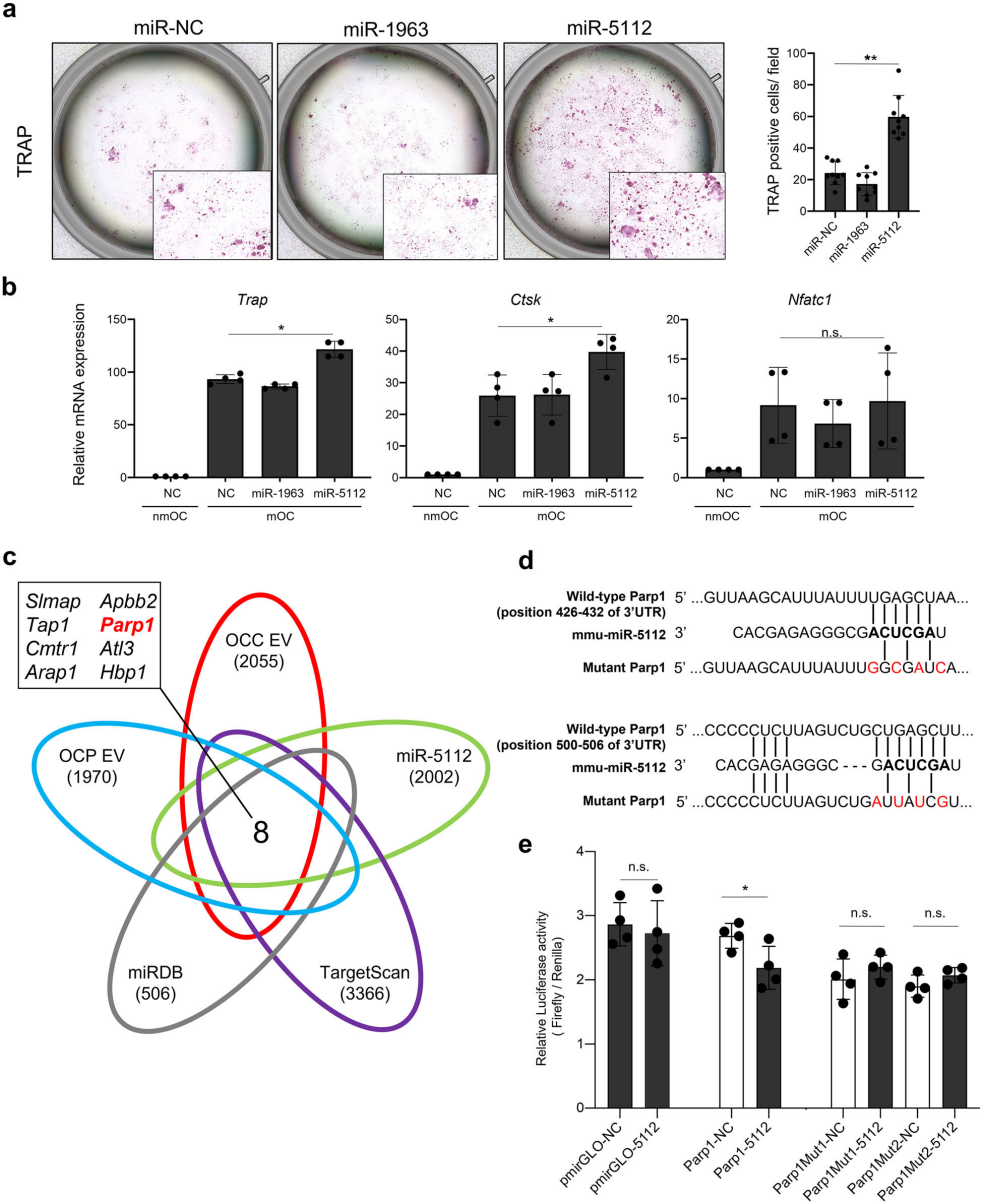
**mmu‐miR‐5112 promote OC activity targeting *Parp1*. (a)** TRAP staining (left panel) and TRAP‐positive cell counts (right panel) in mOCs transfected with representative miR‐mimics. Error bars represent SD of the mean (*n* = 9). **p < 0.01, One‐way ANOVA. RAW 264.7 cells were cultured with RANKL (10 ng/mL) for 5 days after transient transfection with the miR‐mimic. NC: negative control. **(b)** PCR analysis of osteoclastic marker expression in mOCs 72 h after transfection with representative miR‐mimics. nmOC: non‐mOC, NC: negative control. *n* = four biological replicates. Error bars represent SD. *p < 0.05, Student's *t*‐test. n.s., no significance. **(c)** Venn diagram of the overlapping predicted target genes using different online analysis tools (TargetScan and miRDB) and NGS datasets. **(d)** Summary of miR‐5112 target sites and mutated sites in the 3’UTRs of *Parp1*. **(e)** The luciferase reporter assay with the wild‐type and mutant *Parp1* 3’UTR vectors co‐transfected with miR‐5112. Error bars represent the SD of the mean (*n* = 4, *p < 0.05, Student's *t*‐test). n.s., no significance.

To uncover these miRNA impacts on the activity of OC, we conducted the transfection of miR‐1963 and miR‐5112 mimics to RAW 264.7 cells and then induce differentiation in the presence of RANKL. TRAP staining revealed that transfection with the miR‐5112 mimic increased the TRAP‐positive multinuclear cells (Figure 4a). Similar results were obtained by quantitative reverse transcriptase‐PCR (RT‐qPCR) for *Trap*, *Ctsk* and *Nfatc1* which are representative osteolytic markers in OC, although some were not statistically significant (Figure 4b). These data strongly indicated that miR‐5112 promotes OC activity. Next, we identified the predicted target genes using RNA‐seq data together with online miRNA prediction software (TargetScan, miRDB) (Figure 4c). Total RNA was extracted from OCP/OCC/OCN EV‐treated mOCs and miR‐5112/negative control (NC) mimic‐transfected mOC, and NGS was performed. As shown in the Venn diagram, we narrow down eight candidate genes as predicted targets of mmu‐miR‐5112 that were enriched in CAOC EVs (Figure 4c). Among the predicted targets, we focused *on Parp1*, also known as poly ADP‐ribose polymerase 1, which encodes an enzyme called PARP1 that plays a crucial role in various cellular processes, including DNA repair, genomic stability and regulation of gene expression. In rheumatoid arthritis studies, PARP1 has been reported to be strongly associated with inflammatory osteolysis (Mbalaviele et al. 2017). Sensing inflammasomes, cleaved PARP1 and autocrine effect IL‐1β, acting through IL‐1R, promote OC maturation into actively bone‐resorbing phenotype (Mbalaviele et al. 2017). The downregulation of *Parp1* was validated by qRT‐PCR using independent sample sets (Figure S7a). Moreover, we confirmed that the transfection of the miR‐5112 mimic into mOC did not induce cell proliferation as assessed by a cell proliferation assay (Figure S7b). Then, we conducted a luciferase 3’UTR reporter assay to examine whether mmu‐miR‐5112 can directly bind to the 3’UTR of *Parp1*. Assay vectors with sequence variations containing the predicted miR‐5112 target sequences were constructed (Figure 4d). The assay results demonstrated that the wild‐type *Parp1* 3’UTR, but not the other variants, was suppressed by miR‐5112 (Figure 4e). Furthermore, we performed miR‐1963 and miR‐5112 mimic double transfection into mOC to examine whether other miRNAs, including miR‐1963, were equally enriched in CAOC EVs, driving or impairing the effects of miR‐5112 on mOC. As shown in Figure S7c, the miRNA mimic (miR‐mimic) cocktail of miR‐1963 and miR‐5112 also promoted OC activity, indicating that miR‐5112 promotes OC maturation, even in miRNA complexes in CAOC EVs. These findings reveal that EV‐miR‐5112 derived from CAOCs is a contributing factor for OC maturation and *that Parp1* in mOC is directly targeted by mmu‐miR‐5112.

### 3.5 EV‐mmu‐miR‐1963 Suppress OB Activity Targeting *Hoxa1*

**FIGURE 5:**
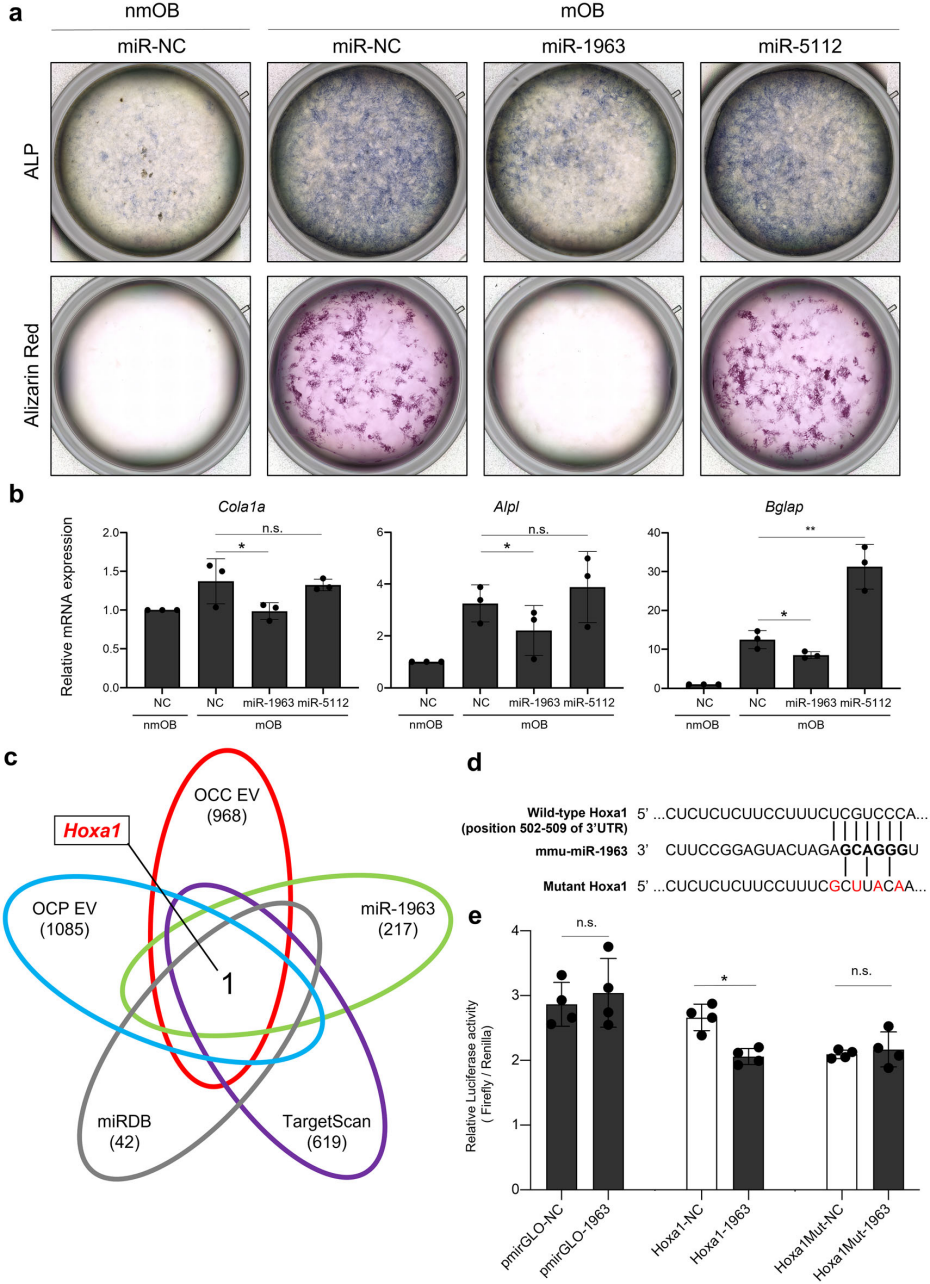
**mmu‐miR‐1963 inhibit OB activity targeting *Hoxa1*. (a)** ALP and Alizarin Red staining of mOBs transfected with representative miR‐mimics. MC3T3‐E1 cells were cultured with or without ascorbic acid and β‐glycerophosphate for 9 and 20 days, respectively, after transient miR‐mimic transfection. nmOB: non‐mOB; NC: negative control. **(b)** RT‐qPCR analysis of osteoblastic marker expression in mOBs transfected with representative miR‐mimics. *Cal1a1* and *Alpl* expression levels were measured at 72 h, and Bglap expression was measured 144 h after transfection. nmOB: non‐mOB; NC: negative control. *n* = 3 biological replicates. Error bars represent SD. *p < 0.05, **p < 0.01, Student's *t*‐test. n.s., no significance. **(c)** Venn diagram of the overlapping predicted target genes using different online analysis tools (TargetScan and miRDB) and NGS datasets. **(d)** Summary of miR‐1963 target sites and mutated sites in the 3’UTRs of *Hoxa1*. **(e)** The luciferase reporter assay with the wild‐type and mutant *Hoxa1* 3’UTR vectors co‐transfected with miR‐1963. Error bars represent the SD of the mean (*n* = 4, *p < 0.05, Student's *t*‐test). n.s., no significance.

Similarly, to investigate the impact of these miRNAs on mOB activity, we performed the transfection with miR‐1963 and miR‐5112 mimics to MC3T3‐E1 cells and then induced differentiation in the presence of ascorbic acid and β‐glycerophosphate. ALP staining on Day 10 and Alizarin Red staining on Day 21 clearly showed that transfection with the miR‐1963 mimic greatly reduced ALP expression levels and calcium deposits in cell cultures, whereas miR‐5112 transfection did not change them compared to the NCs (Figure 5a). Similar results were obtained by RT‐qPCR for *Col1a1*, *Alpl* and *Bglap* which are representative osteoblastic markers of OB (Figure 5b). the transfection of the miR‐1963 mimic significantly suppressed the expression of all three genes. The transfection of the miR‐5112 mimic promoted the gene expression of *Bglap*; however, it did not increase other osteoblastic markers such as *Col1a1* and *Alpl*, nor did it enhance mineralization, as shown in Figure 5a. These data strongly indicated that miR‐1963 suppressed OB activity. Next, we identified the predicted target genes using NGS data together with an online miRNA prediction software (TargetScan, miRDB) (Figure 5c). We prepared total RNA extracted from OCP/OCC/OCN EV‐treated mOBs and miR‐1963/NC mimic‐transfected mOB and then performed NGS analyses. As shown in the Venn diagram, we identified only one gene, *Hoxa1*, which was enriched in CAOC EVs, as a predicted target of mmu‐miR‐1963 (Figure 5c). *Hoxa1* is a member of the Hox gene family and plays a critical role in embryonic development, patterning and differentiation of various tissues. Previous studies suggest that *Hoxa1* may be involved in regulating OB differentiation, particularly in response to cytokine signalling such as IL‐1β (Ma et al. 2019). This decrease in *Hoxa1* expression was validated by qRT‐PCR using independent sample sets (Figure S7d). Moreover, using a cell proliferation assay, we confirmed that miR‐1963 transfection into mOB did not affect mOB viability (Figure S7e). We conducted a luciferase 3’UTR reporter assay to examine whether miR‐1963 can directly bind to the 3’UTR of *Hoxa1*. We constructed assay vectors with several sequence variations including the predicted miR‐1963 target sequences (Figure 5d). The assay results demonstrated that wild‐type *Hoxa1* 3’UTR, but not other variants, was suppressed by miR‐1963 (Figure 5e). Moreover, we performed miR‐1963 and miR‐5112 double transfection into mOB in the same manner for the same reasons as for mOC. As shown in Figure S7f, the miR‐mimic cocktail of miR‐1963 and 5112 also reduced OB activity, indicating that the miR‐1963 suppressive effect on OB mineralization was outstanding among the several candidates enriched in CAOC EVs. These findings revealed that EV‐miR‐1963 is a negative regulator of OB mineralization and *Hoxa1* gene in OB is directly targeted by EV‐miR‐1963.

### 3.6 *Parp1* and *Hoxa1* Knockdown Induce IL‐1β‐Related Reactions in Bone‐Resident Cells

**FIGURE 6:**
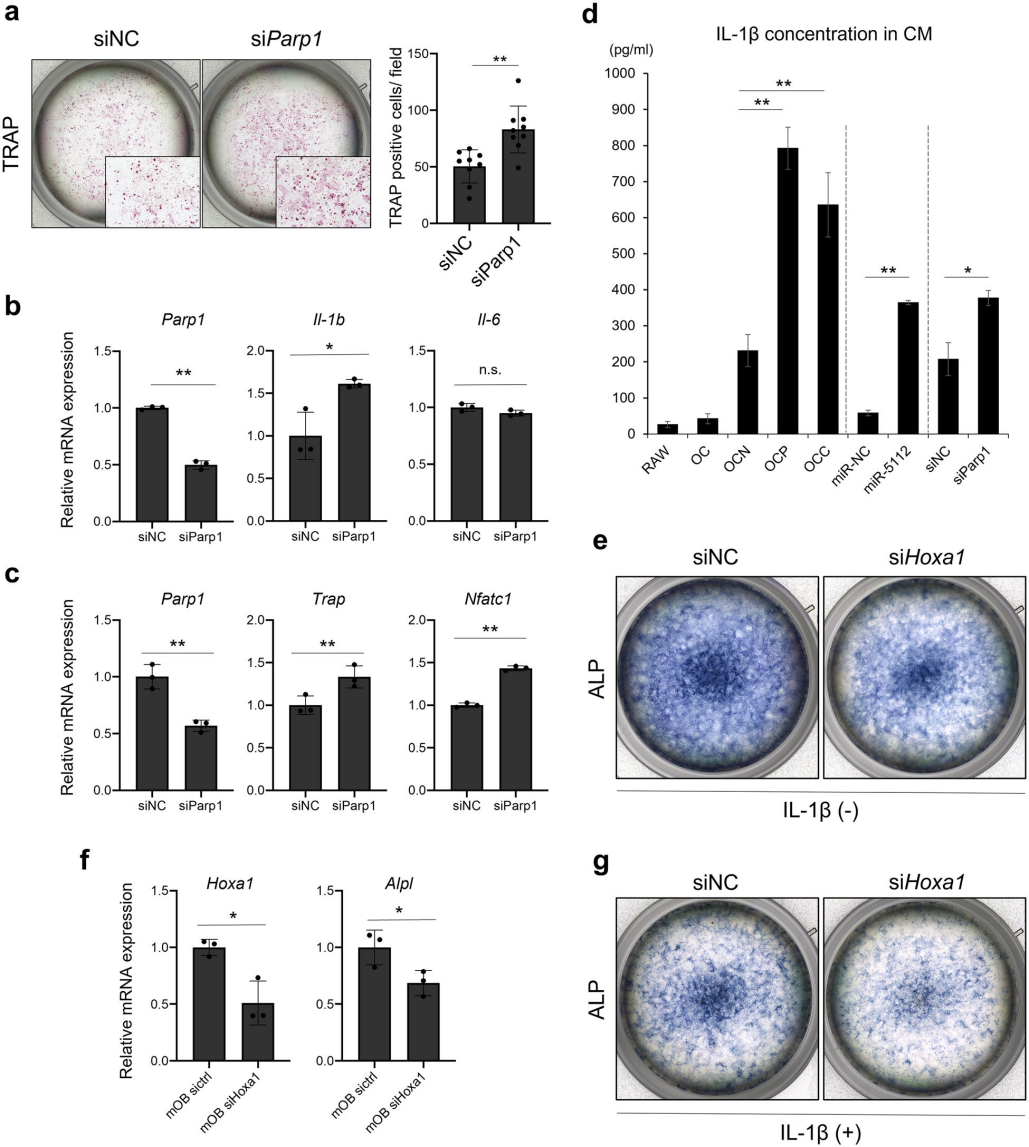
**Effects of *Parp1* knockdown on OC and *Hoxa1* knockdown on OB. (a)** TRAP staining of mOCs transfected with *Parp1* siRNA (left panel) and TRAP‐positive cell counts (right panel). Error bars represent the SD of the mean (*n* = 9). **p < 0.01, Student's *t*‐test. RAW 264.7 cells were cultured with RANKL (10 ng/mL) for 5 days after siRNA transfection. siNC: small interfering negative control. **(b)** effect of *Parp1* knockdown on the expression of interleukin family genes in mOCs. The expression levels of *Parp1, Il‐1b* and *Il‐6* in RAW 264.7 cells in the presence of RANKL were measured 36 h after the transfection. Error bars represent SD. *p < 0.05, **p < 0.01 by Student's *t*‐test. *n* = 3 biological replicates. n.s., no significance. **(c)** effect of *Parp1* knockdown on the expression of osteoclastic marker genes in mOCs. The expression levels of *Parp1*, *Trap* and *Nfatc1* in RAW 264.7 cells in the presence of RANKL were measured 72 h after transfection. Error bars represent SD. *p < 0.05, **p < 0.01 by Student's *t*‐test. *n* = 3 biological replicates. n.s., no significance. **(d)** The concentration of IL‐1β in CM of each OC. Error bars represent SD. *p < 0.05, **p < 0.01 by one‐way ANOVA or Student's *t*‐test. *n* = 3 biological replicates. **(e)** ALP staining of mOBs transfected with *Hoxa1* siRNA without IL‐1β. ALP staining was performed on Day 10. MC3T3‐E1 cells were cultured with ascorbic acid and β‐glycerophosphate for 9 days after transient siRNA transfection. NC: negative control. **(f)** Effect of *Hoxa1* knockdown on expression of the osteoblastic marker *Alpl* in mOBs. The expression levels of *Alpl* in MC3T3‐E1 cells in the presence of ascorbic acid and β‐glycerophosphate were measured 72 h after transfection. Error bars represent SD; *p < 0.05, **p < 0.01, Student's *t*‐test. *n* = 3 biological replicates. NS: no significance. **(g)** ALP staining of mOBs transfected with *Hoxa1* siRNA with IL‐1β. ALP staining was performed on Day 10. MC3T3‐E1 cells were cultured with ascorbic acid and β‐glycerophosphate for 9 days after transient siRNA transfection. NC: negative control.

To further investigate the relevance of *Parp1* in OC maturation and the relevance of *Hoxa1* to OB mineralization, knockdown experiments were performed using short interfering RNA (siRNA). TRAP staining showed that *Parp1* knockdown resulted in OC activation (Figure 6a). The knockdown efficiency of the *Parp1* by siRNAs was confirmed in the RAW 264.7 cells at the gene expression level. As expected, *Parp1* silencing significantly increased *Il‐1b* and OC marker gene expression levels (Figure 6b,c). In contrast, *Il‐6* expression level was not elevated by *Parp1* silencing (Figure 6b). These findings indicated that *Parp1* negatively regulates OC activation and associated with IL‐1β production. To further investigate IL‐1β production in mOC, we measured IL‐1β concentration in CM by ELISA in several OC culture conditions (Figure 6d). The results showed that OC co‐cultured with PCa cell lines, namely, CAOCs secretes much more IL1‐β that OC precursors and NOCs. Likewise, miR‐5112 mimic transfection and *Parp1* silencing increased IL‐1B secretion from OC; however, *Parp1* silencing increased much less than miR‐5112 mimic transfection compared as each control. this indicated that any other miR‐5112 target genes were involved in IL‐1B secretion (Figure 6d). Collectively, these findings indicated that CAOCs produce miR‐5112 enriched EVs mediating *Parp1*‐*Il‐1b* axis in mOC, potentially leading to further inflammatory osteolysis in accordance with IL‐1β secretion from pathologically activated OC. In contrast, we revealed that *Hoxa1* silencing resulted in the reduction of ALP staining levels (Figure 6e). The efficacy of the *Hoxa1* by siRNA was confirmed in MC3T3‐E1 cells at the gene expression level (Figure 6f). Downregulation of ALP expression by *Hoxa1* silencing was validated at the gene expression level (Figure 6f). Finally, we confirmed the suppressive effect on OB activity in the presence of IL‐1β, as *Hoxa1* has been reported to regulate chondrocyte differentiation in response to IL‐1β (Ma et al. 2019). Given that OBs and chondrocytes share certain factors involved in their origin and differentiation, we considered *Hoxa1* as a potential regulator in this context. As a result, the suppressive effect of *Hoxa1* silencing for OB activity was more obvious in the presence of IL‐1β (Figure 6g), indicating that OB might be suppressed by EVs from mOC via miR‐1963‐*Hoxa1* axis in IL‐1β abundant metastatic niche.

### 3.7 Mimic Cocktail of miR‐1963 and miR‐5112 Facilitate Bone Destruction and Tumour Invasion In Vivo

**FIGURE 7:**
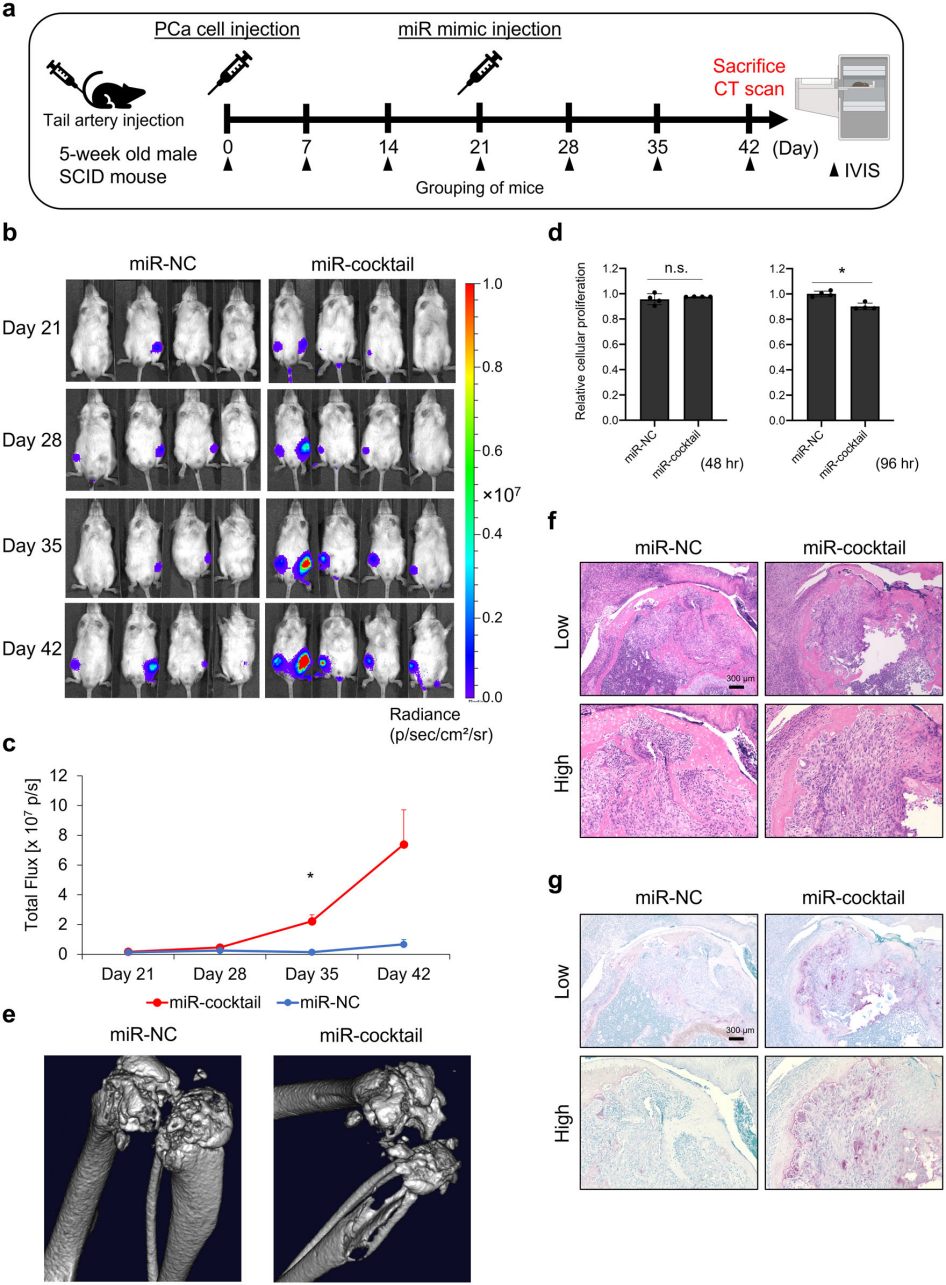
**Mimic cocktail of miR‐1963 and miR‐5112 promote tumour progression and bone destruction in vivo. (a)** Schematic protocol for investigating the effects of representative miR‐mimics on PCa bone metastases. A metastatic PCa mouse model was established using PC3M cells. miR‐1963 and miR‐5112 mimic cocktail and miR‐mimic NC were injected into the caudal artery on Day 21. The mice were sacrificed on Day 42. The images of microcomputed tomography (µCT) of bones are obtained on the same day with sacrifice. **(b)** IVIS imaging of PC3M cells in xenograft mice (*N* = 4). Images acquired on Days 21, 28, 35 and 42. **(c)** Photon counting of PC3M cells in a mouse model of bone metastasis. Error bars represent the SD of the mean (*n* = 4). *p < 0.05 by Student *t*‐test. **(d)** Viability of PC3M cells after transfection with the miR‐1963 and miR‐5112 mimic cocktail. Cell viability was measured using a CCK‐8 assay. Error bars represent the SD of the mean (*n* = 4, *p<0.05, Student's *t*‐test). n.s., no significance. **(e)** Representative images of microcomputed tomography (µCT) of bones in mice injected with miR‐1963 and miR‐5112 mimic cocktail and miR‐mimic NC. **(f)** Representative images of HE‐ stained sections of bone metastatic sites in miR mimic‐injected mice. All images were obtained with an all‐in‐one fluorescence microscope using a 10x objective for low‐ and 20× objective for high‐magnification. Scale bar indicates 300 µm. **(g)** Representative images of TRAP/ALP‐stained sections of bone metastatic sites in miR mimic‐injected mice. These images are serial sections from the same murine bone samples as shown in (f). All images were obtained with an all‐in‐one fluorescence microscope using a 10× objective for low‐ and 20× objective for high‐magnification. Scale bar indicates 300 µm.

Finally, we investigated the role of miR‐1963 and miR‐5112, which were specifically enriched in CAOC EVs, in pathological bone homeostasis and tumour progression. These two miRNAs were almost equally enriched in CAOC EVs, especially in OCC EVs (Table S6). Thus, we mixed miR‐1963 and miR‐5112 mimics and created a mimic cocktail for injection. We used a bone metastasis model established by caudal artery injection of PC3M (Kuchimaru et al. 2018; Yamamoto et al. 2024) and then injected the mimic cocktail via the caudal artery. Bone metastasis was monitored using an IVIS (Figure 7a). In vivo imaging demonstrated that the mimic cocktail significantly increased metastatic tumour volume compared to NC (Figure 7b). On Day 35, the mimic cocktail injection group exhibited more than a 10‐fold increase in bioluminescent expression compared to the NC injection group (Figure 7c). We confirmed that the mimic cocktail did not promote cancer cell proliferation compared with NC in vitro (Figure 7d). The mimic cocktail did not suppress cancer cell proliferation at 48 h but was suppressed at 96 h (Figure 7d). In addition, bone destruction was evaluated using micro‐CT on the day of sacrifice (Figure 7a). Surprisingly, three‐dimensional bone imaging revealed that the mimic cocktail injection caused severe bone destruction (Figure 7e). Histopathological observations showed that TRAP‐positive OCs were significantly increased and severe bone resorption by a large number of OCs was induced in the mimic cocktail injection group compared to the NC injection group, which showed the same level of tumour invasion (Figures 7f,g and S8a,b). These findings suggest that miR‐1963 and miR‐5112, which are enriched in CAOC EVs, induce metastatic tumour growth and bone destruction, probably because of their pathological effects on bone‐resident cells instead of cancer cells.

## 4 Discussion

The bone is the primary destination for metastasis in advanced‐stage PCa. Furthermore, PCa uniquely induces osteogenic bone metastases. However, the mechanisms by which PCa degrades bone and ultimately exhibits osteogenic bone metastases are not clearly understood. Nevertheless, accumulating evidence suggests that the crosstalk between the bone microenvironment and PCa cells is pivotal in unravelling these processes (Tamura et al. 2023). To address this issue, we focused on EVs derived from OCs affected by PCa. In the present study, we found that specific miRNAs within pathological OC‐derived EVs play a pivotal role in disrupting bone homeostasis during PCa bone metastasis. Our data showed that EVs from OCs with PCa cells induce further OC activation while concurrently suppressing OB function by mediating IL‐1β‐related reactions, leading to further bone destruction and metastatic tumour progression.

Although PCa mainly exhibits osteogenic bone metastasis, osteolytic lesions are detected in almost all patients with PCa bone metastasis. Frequent pathological fractures are reportedly induced by these osteolytic lesions (Roudier et al. 2008). Numerous studies have supported the importance of bone‐resorbing OCs, even in osteogenic PCa bone metastases. A pioneering in vivo study revealed that osteogenic bone metastasis development in PCa requires priming of osteoclastic activity (Yonou et al. 2004). This study demonstrated in a murine bone metastasis model using osteogenic PCa cells that OCs first proliferate at the peripheral region of the tumour, followed by cancer cell invasion, which replaces the site with osteoblastic metastatic foci. Our group previously reported that the clinical course of bone metastatic lesions in PCa tended to change from an osteolytic to an osteogenic pattern, and relapse was often accompanied by an increase in osteolytic lesion (Shimazaki et al. 1992). Furthermore, we also reported the diagnostic accuracy of serum bone turnover markers for the detection of bone metastasis in patients with PCa and the usefulness of these markers as predictors of mortality from PCa (Kamiya et al. 2010). Our previous clinical data suggest that both osteoblastic markers (tALP and BAP) and osteoclastic markers (1CTP, TRAP 5b) may be useful in detecting PCa bone metastasis. Surprisingly, among these bone turnover markers, serum 1CTP, which reflects OC activity, was more reliable than the others in detecting bone metastatic spread and predicting survival probability in patients with PCa bone metastasis. These findings suggest that priming bone resorption, that is, OC activation, is essential for the establishment and progression of tumour metastasis. However, the use of the RANKL inhibitor denosumab, a potent inhibitor of bone resorption, did not prolong survival in men with advanced‐stage PCa (Smith et al. 2012). This inconsistency implied the existence of an underlying RANKL‐independent osteolytic mechanism in PCa bone metastasis. Indeed, our previous report showed that serum RANKL levels did not differ among controls, benign prostatic hyperplasia and PCa patients (Kamiya et al. 2011).

In this study, we demonstrated that OC co‐cultured with PCa cells showed insensitivity to the anti‐RANKL antibody denosumab (Figure 1e), which may partially explain why denosumab did not prolong survival in men with advanced‐stage PCa. Our NGS analysis showed that inflammation‐related signalling pathways were upregulated in OC co‐cultured with PCa cells (Figure 2e,f). Among the various inflammatory cytokine‐related genes, *Il‐1b*, *Casp1* and *Tnf* expression levels were clearly elevated in CAOCs compared to NOCs, whereas Il‐6 expression levels were elevated only in OCP cells (Figures 2g and 3a). In the research field of arthritis, IL‐1β, CASP1 and TNF in OC are reported to be key molecules in osteolysis responding to excessive inflammasome activation (Mbalaviele et al. 2017). Cleaved PARP1, which we identified as a candidate target of miR‐5112 enriched in CAOC EVs (Figure 4c), and autocrine effects of IL‐1β are reported to promote OC maturation into pathologically bone‐resorbing cells in arthritis (Mbalaviele et al. 2017). Our data showed that COACs, OCs transfected with miR‐5112 mimic, and *Parp1* knockdown OCs secreted high volume of IL‐1β into CM (Figure 6d). Among various inflammatory cytokines, IL‐1β has been reported to induce pathologically activated OC bearing extremely high levels of resorbing activity (Shiratori et al. 2018). Moreover, previous studies demonstrated that IL‐1β with TNF stimulates OC differentiation by a mechanism independent of RANKL pathway (Kobayashi et al. 2000). IL‐6 with TNF has also been reported to induce osteoclastogenesis independent of the RANKL pathway (Yokota et al. 2021); however, in this study, *Il‐6* expression level was not elevated in OCC cells. Furthermore, our NGS data showed that the expression of *Nfatc1* was not elevated in CAOCs compared to that in NOCs (Figure S4b). Likewise, the expression of *Nfatc1* was not elevated in OCs transfected with miR‐5112 enriched in CAOC EVs (Figure 4b). NFATc1, also known as nuclear factor of activated T‐cells, cytoplasmic 1, is a transcription factor that represents a master regulator of OC differentiation, functioning downstream of RANKL signalling. Takayanagi et al. (2002) Collectively, these findings suggested that PARP1‐IL‐1β axis contributes the pathological phenotype as CAOCs independent of RANKL signalling and this phenotype change may be disseminated to other OCs by EV transfer. We focused on *Parp1* among the eight genes identified as potential targets of miR‐5112 based on a review of previous literature. However, the other seven genes may also be involved in IL‐1β through complex interactions, and further research is needed to clarify this point.

Ever since Paget proposed the “seed and soil theory” back in 1889 (Paget 1889), numerous studies have demonstrated that the surrounding environment is essential for cancer progression. PCa bone metastasis results from the interactions between cancer cells and the surrounding bone microenvironment. Conventionally, cytokines, chemokines, and adhesion molecules have been considered as representative mediators of tumour‐host bone microenvironment interactions; however, recent investigations have revealed that EVs are also powerful mediators shuttling various bioactive molecules (Nishida‐Aoki and Ochiya 2024). For instance, several studies have demonstrated that EVs from PCa cells at the primary site modulate the host microenvironment to form a pre‐metastatic niche and may govern bone metastatic tropism in the early stages of bone metastasis. EV‐mediated transfer of the pyruvate kinase M2 (PKM2) protein from primary PCa cells to bone marrow stromal cells has been reported to promote tumour proliferation via upregulation of CXCL12, a chemokine associated with cell migration (Dai et al. 2019). Another study showed that EV‐miR‐92a‐1‐5p from primary PCa downregulates type I collagen expression by directly targeting COL1A1, thereby promoting OC differentiation and inhibiting OB differentiation, thus directly degrading the bone ECM. In this study, EVs produced by osteogenic (MDA‐PCa 2b), osteolytic (PC3) or mixed (C4‐Siegel et al. 2023)) human PCa cells were used; EVs from all three types of PCa stimulated osteoclastogenesis in vitro and provoked osteolysis in vivo (Yu et al. 2021). These findings provide evidence to support EV‐mediated communication for creating an osteolytic pre‐metastatic niche for PCa tumours to spread to the bone.

Primary PCa cells and disseminated PCa cells release EVs, which disrupt the bone remodelling balance. Excessive OB activity induced by metastatic PCa cells results in an osteogenic phenotype. EV‐miR‐141‐3p derived from osteoblastic MDA‐PCa 2b cells promotes OB activity in vitro and osteoblastic bone metastasis in vivo (Ye et al. 2017). Another study showed that hsa‐miR‐940, which is highly enriched in osteoblastic PCa cells, promotes osteogenic BM‐MSCs in vitro, targets ARHGAP1 and FAM134A, and induces extensive osteoblastic lesions in vivo (Hashimoto et al. 2018). Conversely, the excessive OC activity induced by metastatic PCa leads to an osteolytic phenotype. CDCP1, located on EVs derived from metastatic PCa, facilitates OC formation (Urabe et al. 2023). These studies focused on the roles of EVs from PCa cells in a single type of bone‐resident cell. Other studies have focused on EVs derived from bone‐resident cells. For example, one study showed that OB‐derived EVs upregulate SERPINA3 and LCN2 in PCa cells, contributing to the osteoblastic phenotype and preventing further tumour spread (Ito et al. 2023). The importance of OB‐derived EVs has been previously reported in the field of breast cancer. It has been suggested that OB‐derived EVs regulate the proliferation of cancer cells through the miR‐148‐3p/ERK2 axis (Shupp et al. 2021). However, EV‐mediated crosstalk between bone‐resident cells was not considered in these studies. To the best of our knowledge, no previous study has investigated the effects of OC‐derived EVs on other bone‐resident cells in the presence of PCa cells. Therefore, in this study, we did not focus on EVs from PCa cells, but on EVs from pathological OCs and their effects on other OCs and OBs.

Recently, OC‐derived EVs have been reported to regulate OB activity during normal homeostasis (Ikebuchi et al. 2018; Sun et al. 2016). Therefore, we hypothesized that pathological OC‐derived EVs play crucial roles in bone metastasis. We believed that EVs from OCs affected by osteogenic PCa are crucial cues for the transition from the osteolytic to the osteogenic phase in the process of PCa bone metastasis. To confirm this hypothesis, we used two types of osteolytic PC3M and osteogenic C4‐2B cells to assess gene expression in OC and the characteristics of EVs from OCs co‐cultured with each type of PCa cell. Unexpectedly, OC gene expression was similar between the two types of OCs (Figure 2b–d). Thus, osteolytic and osteogenic PCa had almost the same effect on the OC phenotype. Similarly, EVs derived from each type of OC had almost the same miRNA profiles and the same effects on mOC and mOB (Figure 3d–f). These findings suggest that pathological OC‐derived EVs may play a crucial role not as determinants of the osteogenic metastatic phenotype, but as drivers of bone destruction and subsequent tumour progression, even in osteogenic PCa bone metastasis. Our data indicate that the switch from the osteolytic to the osteoblastic phase is caused by other factors. Indeed, OB co‐cultured with PCa cells showed high expression levels of osteoblastic marker genes (Figure S9a,b), although PCa EVs did not promote OB activity (Figure S9c,d). These findings imply that other secretomes, such as tumour‐secreted cytokines, determine the osteogenic phenotype of PCa bone metastases. We showed that some secretomes from PCa cells changed OC and OB characteristics but did not carry out further investigation of PCa‐derived factors. Because PCa bone metastasis is a highly complex and fluctuating system regulated by numerous factors, further studies are required to elucidate the molecular mechanisms underlying the osteogenic phenotype of PCa bone metastasis.

Although this evidence elucidates novel EV‐mediated intercellular communication networks among bone‐resident cells in the bone tumour microenvironment, there are a few limitations to the present study. One of these is that RAW 264.7, used in this study as an OC precursor, is a murine immortalized cell line. Accordingly, mmu‐miR‐1963 and mmu‐miR‐5112 in EVs from pathological OCs are mouse‐specific miRNAs. Human‐derived cells are desirable; however, they did not stably differentiate into mature OCs in all experiments, even under the same conditions. RAW 264.7 cells are able to stably differentiate into mature OCs only in the presence of RANKL, whereas other human‐derived cells such as THP‐1 cells, human peripheral mononuclear cells, or induced pluripotent stem cells require several factors such as M‐CSF in addition to RANKL and complicated steps to differentiate into mature OCs. It is difficult to obtain almost the same differentiation levels of OCs in every experiment using a complicated differentiation protocol with several types of reagents. Also, primary murine bone marrow derived macrophages were not used in this study due to ethical considerations. To investigate the functions of EVs from each type of OC, a large quantity of EVs was required. Obtaining this amount would have necessitated sacrificing a significant number of mice to generate a sufficient number of primary cells. Therefore, we used RAW 264.7 cells as OC precursors to simplify the protocol for the stable collection and analysis of EVs from mature OCs co‐cultured with PCa.

Another limitation was that we did not inject EVs from pathological OCs in the animal studies. Generally, a large number of EVs must be prepared to investigate their effects in vivo. Because we used a co‐culture system to collect pathological OC‐derived EVs, we could not prepare such a large number of EVs. Owing to this technical difficulty, our data did not provide direct evidence that CAOC EVs modulate bone‐resident cells to establish an osteolytic niche and promote tumour progression in vivo.

Regardless, the identified targets of miRNAs enriched in CAOC EVs, such as *Parp1* in OCs and *Hoxa1* in OBs, are preserved in human bone‐resident cells. Indeed, PARP1 and HOXA1 have been reported as key molecules in IL‐1β‐related inflammatory osteolysis and chondrocyte degradation, respectively, in human diseases such as arthritis. Although it is evident that bone resorption by OCs is an essential event for bone metastasis, even in osteogenic PCa, the reason why OC‐targeted therapy does not prevent tumour invasion is unclear. We have provided a potential explanation for this clinical issue. The dominant PCa cells corrupt OCs and induce an inflammatory phenotype. Pathological OCs release EVs that mediate inflammatory reactions in bone‐resident cells, leading to further bone destruction and tumour progression.

## Author Contributions

**Takahiro Ochiya**: Resources (lead); supervision (lead); writing ‐ original draft (supporting). **Takaaki Tamura**: Conceptualization (lead); data curation (lead); methodology (lead); writing ‐ original draft (lead); writing ‐ review and editing (lead). **Tomofumi Yamamoto**: Data curation (supporting); formal analysis (supporting); investigation (supporting); writing ‐ original draft (supporting). **Akiko Kogure**: Data curation (supporting). **Yusuke Yoshioka**: Formal analysis (supporting); software (supporting). **Yusuke Yamamoto**: Data curation (supporting); formal analysis (supporting); methodology (supporting); visualization (supporting). **Shinichi Sakamoto**: Supervision (supporting). **Tomohiko Ichikawa**: Supervision (supporting).

## Conflicts of Interest

The authors declare no competing interest.

## Supporting information



Supporting Information

Supporting Information

## Data Availability

The NGS data supporting this study are available from the NCBI for Biotechnology Information database. The GEO accession number for our NGS data was GSE (268422, 268548, 268549, 268550). All other relevant data are available within the article file or supplementary information or are available from the authors upon reasonable request.

## References

[jev270091-bib-0001] Boyle, W. J. , W. S. Simonet , and D. L Lacey . 2003. “Osteoclast Differentiation and Activation.” Nature 423, no. 6937: 337–342. 12748652 10.1038/nature01658

[jev270091-bib-0002] Bray, F. , M. Laversanne , H. Sung , et al. 2024. “Global Cancer Statistics 2022: GLOBOCAN Estimates of Incidence and Mortality Worldwide for 36 Cancers in 185 Countries.” CA: A Cancer Journal for Clinicians 74, no. 3: 229–263. 38572751 10.3322/caac.21834

[jev270091-bib-0003] Dai, J. , J. Escara‐Wilke , J. M. Keller , et al. 2019. “Primary Prostate Cancer Educates Bone Stroma Through Exosomal Pyruvate Kinase M2 to Promote Bone Metastasis.” Journal of Experimental Medicine 216, no. 12: 2883–2899. 31548301 10.1084/jem.20190158 PMC6888980

[jev270091-bib-0004] Hashimoto, K. , H. Ochi , S. Sunamura , et al. 2018. “Cancer‐Secreted hsa‐miR‐940 Induces an Osteoblastic Phenotype in the Bone Metastatic Microenvironment via Targeting ARHGAP1 and FAM134A.” PNAS 115, no. 9: 2204–2209. 29440427 10.1073/pnas.1717363115 PMC5834702

[jev270091-bib-0005] Ikebuchi, Y. , S. Aoki , M. Honma , et al. 2018. “Coupling of Bone Resorption and Formation by RANKL Reverse Signalling.” Nature 561, no. 7722: 195–200. 30185903 10.1038/s41586-018-0482-7

[jev270091-bib-0006] Ito, K. , T. Yamamoto , Y. Hayashi , et al. 2023. “Osteoblast‐Derived Extracellular Vesicles Exert Osteoblastic and Tumor‐Suppressive Functions via SERPINA3 and LCN2 in Prostate Cancer.” Molecular Oncology 17, no. 10: 2147–2167. 37408474 10.1002/1878-0261.13484 PMC10552899

[jev270091-bib-0007] Kamiya, N. , H. Suzuki , T. Endo , et al. 2011. “Significance of Serum Osteoprotegerin and Receptor Activator of Nuclear Factor κB Ligand in Japanese Prostate Cancer Patients With Bone Metastasis.” International Journal of Clinical Oncology 16, no. 4: 366–372. 21327451 10.1007/s10147-011-0193-7

[jev270091-bib-0008] Kamiya, N. , H. Suzuki , M. Yano , et al. 2010. “Implications of Serum Bone Turnover Markers in Prostate Cancer Patients With Bone Metastasis.” Urology 75, no. 6: 1446–1451. 20206975 10.1016/j.urology.2009.11.049

[jev270091-bib-0009] Khosla, S. 2001. “Minireview: The OPG/RANKL/RANK System.” Endocrinology 142, no. 12: 5050–5055. 11713196 10.1210/endo.142.12.8536

[jev270091-bib-0010] Kobayashi, K. , N. Takahashi , E. Jimi , et al. 2000. “Tumor Necrosis Factor α Stimulates Osteoclast Differentiation by a Mechanism Independent of the Odf/Rankl–Rank Interaction.” Journal of Experimental Medicine 191, no. 2: 275–286. 10637272 10.1084/jem.191.2.275 PMC2195746

[jev270091-bib-0011] Kuchimaru, T. , N. Kataoka , K. Nakagawa , et al. 2018. “A Reliable Murine Model of Bone Metastasis by Injecting Cancer Cells Through Caudal Arteries.” Nature Communications 9, no. 1: 2981. 10.1038/s41467-018-05366-3 PMC6065368 30061695

[jev270091-bib-0012] Lee, J. , B. S. Hong , H. S. Ryu , et al. 2017. “Transition Into inflammatory Cancer‐Associated Adipocytes in Breast Cancer Microenvironment Requires microRNA Regulatory Mechanism.” PLOS ONE 12, no. 3: e0174126. 28333977 10.1371/journal.pone.0174126 PMC5363867

[jev270091-bib-0013] Li, D. , J. Liu , B. Guo , et al. 2016. “Osteoclast‐Derived Exosomal miR‐214‐3p Inhibits Osteoblastic Bone Formation.” Nature Communications 7: 10872. 10.1038/ncomms10872 PMC4786676 26947250

[jev270091-bib-0014] Ma, Y. , Y. Wu , J. Chen , et al. 2019. “miR‐10a‐5p Promotes Chondrocyte Apoptosis in Osteoarthritis by Targeting HOXA1.” Molecular Therapy Nucleic Acids 14: 398. 30731321 10.1016/j.omtn.2018.12.012 PMC6365368

[jev270091-bib-0015] Maeda, H. , M. Koizumi , K. Yoshimura , T. Yamauchi , T. Kawai , and E. Ogata . 1997. “Correlation Between Bone Metabolic Markers and Bone Scan in Prostatic Cancer.” Journal of Urology 157, no. 2: 539–543. 8996351

[jev270091-bib-0016] Mbalaviele, G. , D. V. Novack , G. Schett , and S. L Teitelbaum . 2017. “Inflammatory Osteolysis: A Conspiracy Against Bone.” Journal of Clinical Investigation 127, no. 6: 2030–2039. 28569732 10.1172/JCI93356 PMC5451216

[jev270091-bib-0017] Nishida‐Aoki, N. , and T. Ochiya . 2024. “Impacts of Tissue Context on Extracellular Vesicles‐Mediated Cancer–Host Cell Communications.” Cancer Science 115, no. 6: 1726–1737. 38532284 10.1111/cas.16161 PMC11145126

[jev270091-bib-0018] Nørgaard, M. , A. Ø. Jensen , J. B. Jacobsen , K. Cetin , J. P. Fryzek , and H. T Sørensen . 2010. “Skeletal Related Events, Bone Metastasis and Survival of Prostate Cancer: A Population Based Cohort Study in Denmark (1999 to 2007).” Journal of Urology 184, no. 1: 162–167. 20483155 10.1016/j.juro.2010.03.034

[jev270091-bib-0019] Ono, M. , N. Kosaka , and N. Tominaga , et al. 2014. “Exosomes From Bone Marrow Mesenchymal Stem Cells Contain a microRNA That Promotes Dormancy in Metastatic Breast Cancer Cells.” Science signaling 7, no. 332: ra63. 24985346 10.1126/scisignal.2005231

[jev270091-bib-0020] Paget, S. 1889. “The Distribution of Secondary Growths in Cancer of the Breast.” Lancet 133, no. 3421: 571–573. 2673568

[jev270091-bib-0021] Peinado, H. , M. Alečković , S. Lavotshkin , et al. 2012. “Melanoma Exosomes Educate Bone Marrow Progenitor Cells Toward a Pro‐Metastatic Phenotype Through MET.” Nature Medicine 18, no. 6: 883–891. 10.1038/nm.2753 PMC3645291 22635005

[jev270091-bib-0022] Raposo, G. , and W. Stoorvogel . 2013. “Extracellular Vesicles: Exosomes, Microvesicles, and Friends.” Journal of Cell Biology 200, no. 4: 373–383. 23420871 10.1083/jcb.201211138 PMC3575529

[jev270091-bib-0023] Roodman, G. D. 2004. “Mechanisms of Bone Metastasis.” New England Journal of Medicine 350, no. 16: 1655–1664. 15084698 10.1056/NEJMra030831

[jev270091-bib-0024] Roudier, M. P. , C. Morrissey , L. D. True , C. S. Higano , R. L. Vessella , and S. M Ott . 2008. “Histopathologic Assessment of Prostate Cancer Bone “Osteoblastic” Metastases.” Journal of Urology 180, no. 3: 1154–1160. 18639279 10.1016/j.juro.2008.04.140 PMC2992811

[jev270091-bib-0025] Shimazaki, J. , T. Higa , S. Akimoto , M. Masai , and S. Isaka . 1992. “Clinical Course of Bone Metastasis From Prostatic Cancer Following Endocrine Therapy: Examination With Bone X‐Ray.” Prostate Cancer and Bone Metastasis 324: 269–275. 10.1007/978-1-4615-3398-6_29 1283501

[jev270091-bib-0026] Shiratori, T. , Y. Kyumoto‐Nakamura , A. Kukita , et al. 2018. “IL‐1β Induces Pathologically Activated Osteoclasts Bearing Extremely High Levels of Resorbing Activity: A Possible Pathological Subpopulation of Osteoclasts, Accompanied by Suppressed Expression of Kindlin‐3 and Talin‐1.” Journal of Immunology 200, no. 1: 218–228. 10.4049/jimmunol.1602035 29141864

[jev270091-bib-0027] Shupp, A. B. , M. Neupane , L. C. Agostini , G. Ning , J. R. Brody , and K. M Bussard . 2021. “Stromal‐Derived Extracellular Vesicles Suppress Proliferation of Bone Metastatic Cancer Cells Mediated by ERK2.” Molecular Cancer Research 19, no. 10: 1763–1777. 34021072 10.1158/1541-7786.MCR-20-0981 PMC8492519

[jev270091-bib-0028] Siegel, R. L. , K. D. Miller , N. S. Wagle , and A. Jemal . 2023. “Cancer Statistics, 2023.” CA: A Cancer Journal for Clinicians 73, no. 1: 17–48. 36633525 10.3322/caac.21763

[jev270091-bib-0029] Smith, M. R. , F. Saad , R. Coleman , et al. 2012. “Denosumab and Bone‐Metastasis‐Free Survival in Men With Castration‐Resistant Prostate Cancer: Results of a Phase 3, Randomised, Placebo‐Controlled Trial.” Lancet 379, no. 9810: 39–46. 22093187 10.1016/S0140-6736(11)61226-9 PMC3671878

[jev270091-bib-0030] Sturge, J. , M. P. Caley , and J. Waxman . 2011. “Bone Metastasis in Prostate Cancer: Emerging Therapeutic Strategies.” Nature Reviews Clinical oncology 8, no. 6: 357–368. 10.1038/nrclinonc.2011.67 21556025

[jev270091-bib-0031] Sun, W. , C. Zhao , Y. Li , et al. 2016. “Osteoclast‐Derived microRNA‐Containing Exosomes Selectively Inhibit Osteoblast Activity.” Cell Discovery 2: 16015. 27462462 10.1038/celldisc.2016.15 PMC4886818

[jev270091-bib-0032] Takayanagi, H. , S. Kim , T. Koga , et al. 2002. “Induction and Activation of the Transcription Factor NFATc1 (NFAT2) Integrate RANKL Signaling in Terminal Differentiation of Osteoclasts.” Developmental Cell 3, no. 6: 889–901. 12479813 10.1016/s1534-5807(02)00369-6

[jev270091-bib-0033] Tamura, T. , Y. Yoshioka , S. Sakamoto , T. Ichikawa , and T. Ochiya . 2023. “Extracellular Vesicles in Bone Homeostasis: Key Roles of Physiological and Pathological Conditions.” Journal of Bone and Mineral Metabolism 41, no. 3: 345–357. 35943593 10.1007/s00774-022-01362-2

[jev270091-bib-0034] Tominaga, N. , N. Kosaka , M. Ono , et al. 2015. “Brain Metastatic Cancer Cells Release microRNA‐181c‐Containing Extracellular Vesicles Capable of Destructing Blood–Brain Barrier.” Nature Communications 6, no. 1: 6716. 10.1038/ncomms7716 PMC4396394 25828099

[jev270091-bib-0035] Urabe, F. , N. Kosaka , Y. Yamamoto , et al. 2023. “Metastatic Prostate Cancer‐Derived Extracellular Vesicles Facilitate Osteoclastogenesis by Transferring the CDCP1 Protein.” Journal of Extracellular Vesicles 12, no. 3: 12312. 36880252 10.1002/jev2.12312 PMC9989745

[jev270091-bib-0036] Wang, Y. , H. Chang , H. Liu , et al. 2018. “mmu‐miR‐1963 Negatively Regulates the Ameloblast Differentiation of LS8 Cell Line by Directly Targeting Smoc2 3'UTR.” Experimental Cell Research 362, no. 2: 444–449. 29233684 10.1016/j.yexcr.2017.12.008

[jev270091-bib-0037] Webber, J. , R. Steadman , M. D. Mason , Z. Tabi , and A. Clayton . 2010. “Cancer Exosomes Trigger Fibroblast to Myofibroblast Differentiation.” Cancer Research 70, no. 23: 9621–9630. 21098712 10.1158/0008-5472.CAN-10-1722

[jev270091-bib-0038] Welsh, J. A. , D. C. I. Goberdhan , and L. O'Driscoll , et al. 2024. “Minimal Information for Studies of Extracellular Vesicles (MISEV2023): From Basic to Advanced Approaches.” Journal of Extracellular Vesicles 13, no. 2: e12404. 38326288 10.1002/jev2.12404 PMC10850029

[jev270091-bib-0039] Yamamoto, T. , J. Nakayama , F. Urabe , et al. 2024. “Aberrant Regulation of Serine Metabolism Drives Extracellular Vesicle Release and Cancer Progression.” Cell Reports 43, no. 8: 114517. 39024098 10.1016/j.celrep.2024.114517

[jev270091-bib-0040] Yáñez‐Mó, M. , P. R. M. Siljander , Z. Andreu , et al. 2015. “Biological Properties of Extracellular Vesicles and Their Physiological Functions.” Journal of Extracellular Vesicles 4: 1–60. 10.3402/jev.v4.27066 PMC4433489 25979354

[jev270091-bib-0041] Ye, Y. , S. L. Li , Y. Y. Ma , et al. 2017. “Exosomal miR‐141‐3p Regulates Osteoblast Activity to Promote the Osteoblastic Metastasis of Prostate Cancer.” Oncotarget 8, no. 5: 94834–94849. 29212270 10.18632/oncotarget.22014 PMC5706916

[jev270091-bib-0042] Yokoi, A. , Y. Yoshioka , and Y. Yamamoto , et al. 2017. “Malignant Extracellular Vesicles Carrying MMP1 mRNA Facilitate Peritoneal Dissemination in Ovarian Cancer.” Nature Communications 8, no. 1: 14470. 10.1038/ncomms14470 PMC5343481 28262727

[jev270091-bib-0043] Yokota, K. , K. Sato , T. Miyazaki , et al. 2021. “Characterization and Function of Tumor Necrosis Factor and Interleukin‐6–Induced Osteoclasts in Rheumatoid Arthritis.” Arthritis Rheumatol Hoboken Nj 73, no. 7: 1145–1154. 10.1002/art.41666 PMC8361923 33512089

[jev270091-bib-0044] Yonou, H. , A. Ochiai , M. Goya , et al. 2004. “Intraosseous Growth of Human Prostate Cancer in Implanted Adult Human Bone: Relationship of Prostate Cancer Cells to Osteoclasts in Osteoblastic Metastatic Lesions.” Prostate 58, no. 4: 406–413. 14968441 10.1002/pros.10349

[jev270091-bib-0045] Yoshioka, Y. , N. Kosaka , Y. Konishi , et al. 2014. “Ultra‐Sensitive Liquid Biopsy of Circulating Extracellular Vesicles Using ExoScreen.” Nature Communications 5, no. 1: 3591. 10.1038/ncomms4591 PMC3988821 24710016

[jev270091-bib-0046] Yu, L. , B. Sui , and W. Fan , et al. 2021. “Exosomes Derived From Osteogenic Tumor Activate Osteoclast Differentiation and Concurrently Inhibit Osteogenesis by Transferring COL1A1‐Targeting miRNA‐92a‐1‐5p.” Journal of Extracellular Vesicles 10, no. 3: e12056. 33489015 10.1002/jev2.12056 PMC7812369

