# Supplementary material for: Extracellular Vesicles From Prostate Cancer‐Corrupted Osteoclasts Drive a Chain Reaction of Inflammatory Osteolysis and Tumour Progression at the Bone Metastatic Site
Source: J Extracell Vesicles. 2025 Jun 23;14(6):e70091. doi: 10.1002/jev2.70091 (PMC12183380; doi:10.1002/jev2.70091)
Supplement: Supplementary file 2 — Supporting Information [file JEV2-14-e70091-s002.docx]

**Supplementary figures**

**
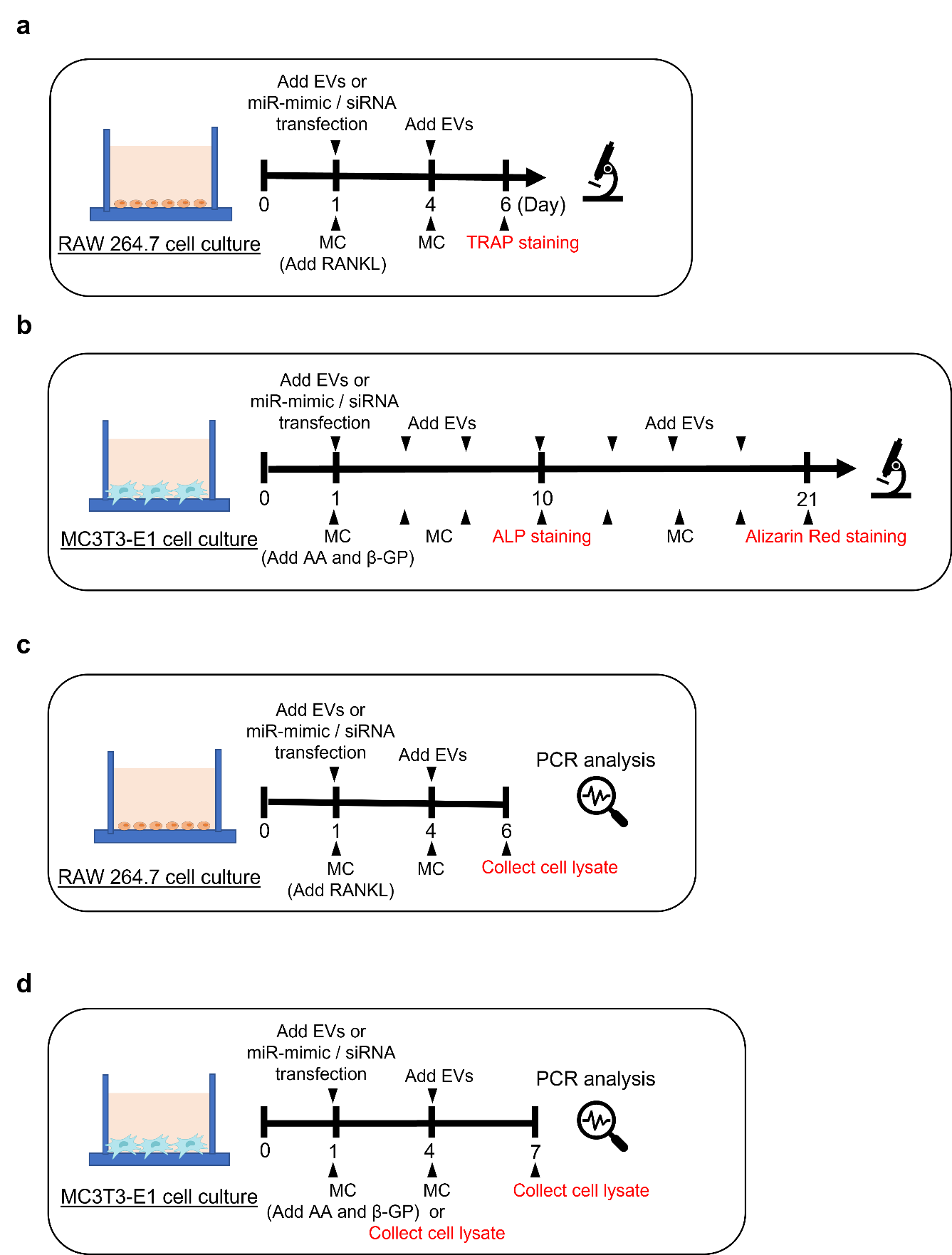
**

**Supplementary figure 1. (a)** Schematic protocol for osteoclastogenesis assay and TRAP staining. **(b)** Schematic protocol for osteoblastogenesis assay and ALP / Alizarin red staining. **(c)** Schematic protocol for osteoclastogenesis assay and PCR analysis. **(d)** Schematic protocol for osteoblastogenesis assay and PCR analysis.　MC: medium change.

**
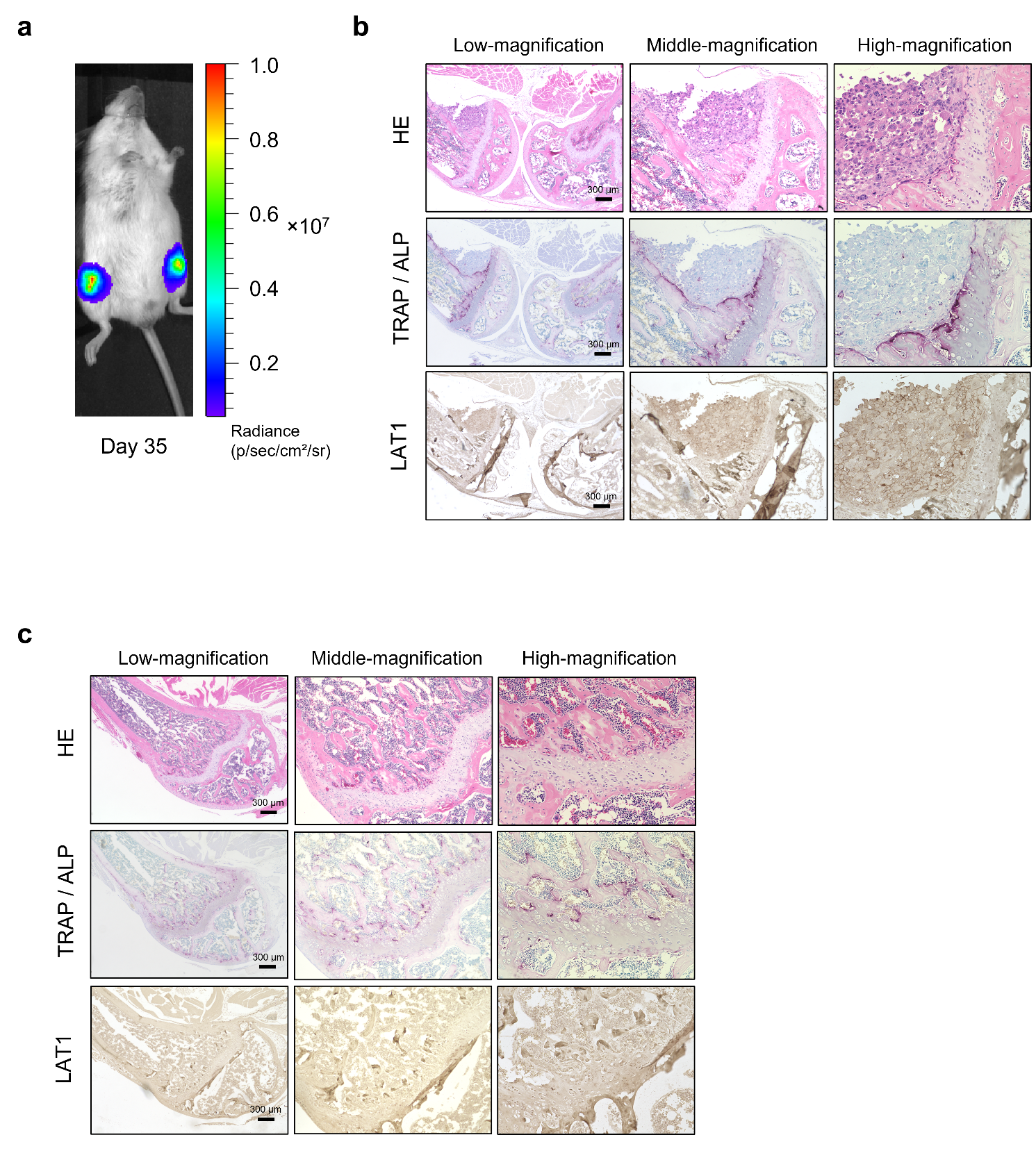
**

**Supplementary figure 2. (a)** IVIS image of another PCa-transplanted xenograft mouse on day 35. Tumors in the hind limbs were evaluated based on the photon radiance of cancer cell bioluminescence. **(b)** Images of HE-, osteoclastic and osteoblastic marker TRAP/ALP-, and cancer specific marker LAT1- stained sections of bone metastatic sites in xenografted mice PCa cells shown in (a). The metastatic tumor developed in the tibia. Scale bar indicates 300 μm. All images were obtained with an all-in-one fluorescence microscope using a 4x objective for low-magnification image, 10x objective for middle-magnification image, and 20x objective for high-magnification image. **(c)** Images of HE-, TRAP/ALP-, and LAT1- stained sections of another non-metastatic limb in the xenograft mouse shown in Fig. 1a. Scale bar indicates 300 μm. All images were obtained with an all-in-one fluorescence microscope using a 4x objective for low-magnification image, 10x objective for middle-magnification image, and 20x objective for high-magnification image.


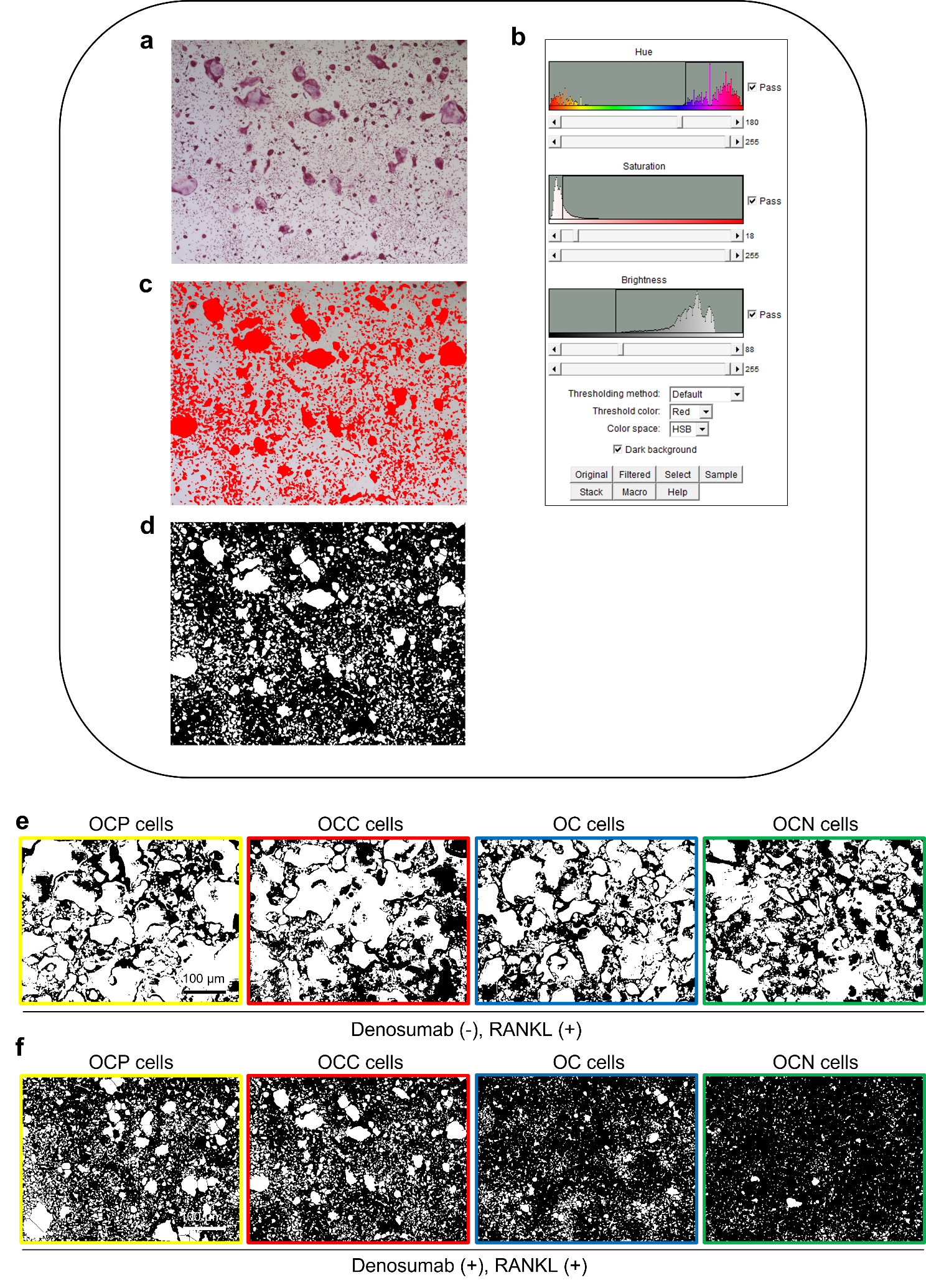


**Supplementary figure 3.** Description of the imageJ software analysis procedure: **(a)** original TRAP staining image, **(b)** analogous color level histogram, **(c)** analyzed picture and **(d)** binary image of TRAP staining. The regions displayed in white within the figure are identified as TRAP-positive cells through image analysis. **(e)** Representative TRAP staining binary image of each OC. Bars represent 100 μm. (**f)** Representative TRAP staining binary image of each OC treated with denosumab at the concentration of 10 μg/mL. Bars represent 100 μm. For more information, please refer to the Supplementary materials and methods.


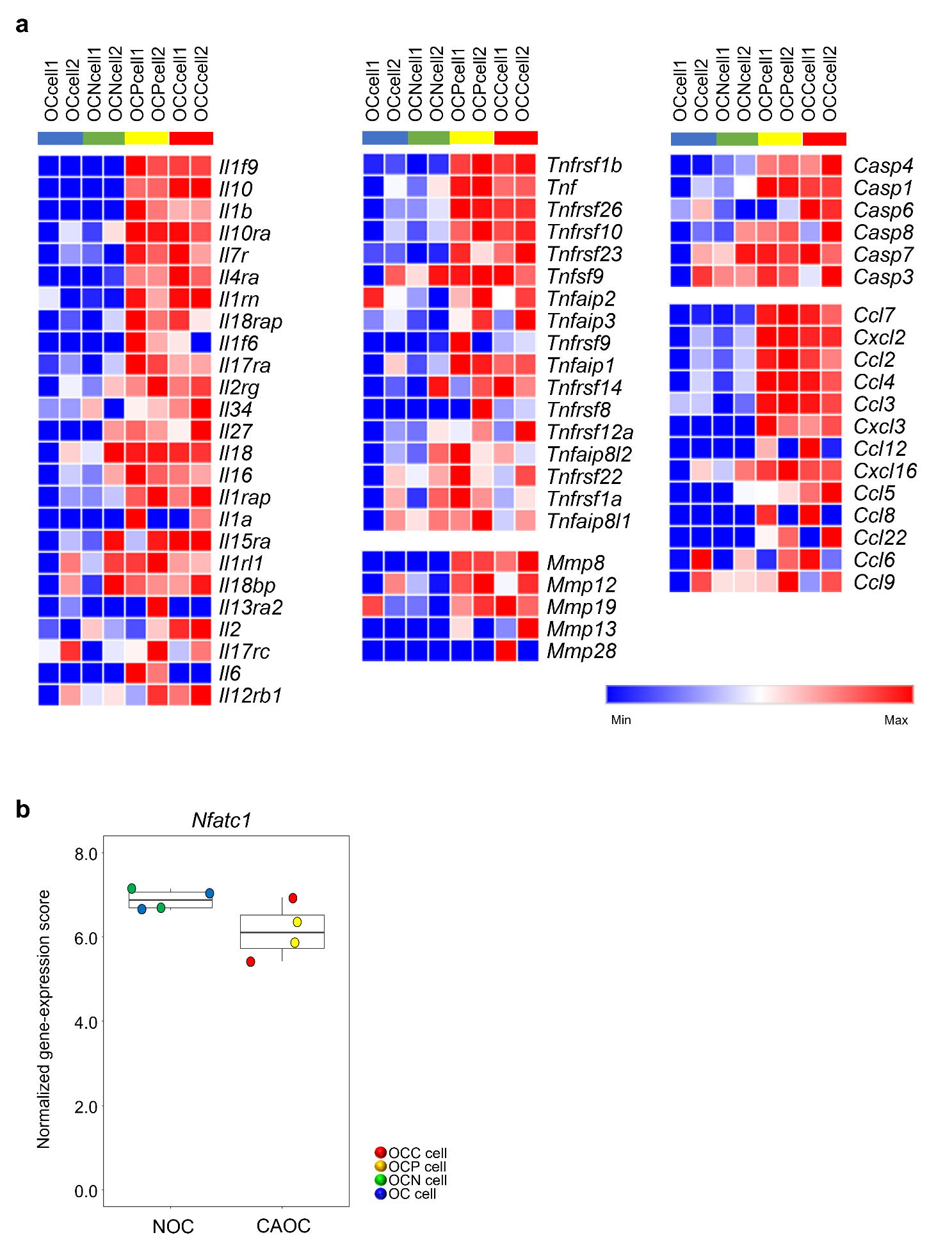


**Supplementary figure 4. (a)** Heatmap showing the selected gene sets. Interleukin cytokine, caspase, MMP, TNF, CCL, and CXCL family genes are also presented. **(b)** Expression levels of *Nfatc1* in NGS datasets. OCs co-cultured with blank inserts (OC cells) or PNT2 (OCN cells) and OCs co-cultured with PC3M (OCP cells) or C4-2B (OCC cells) are presented. The blue, green, yellow, and red dots represent the OC, OCN, OCP, and OCC cell data, respectively.


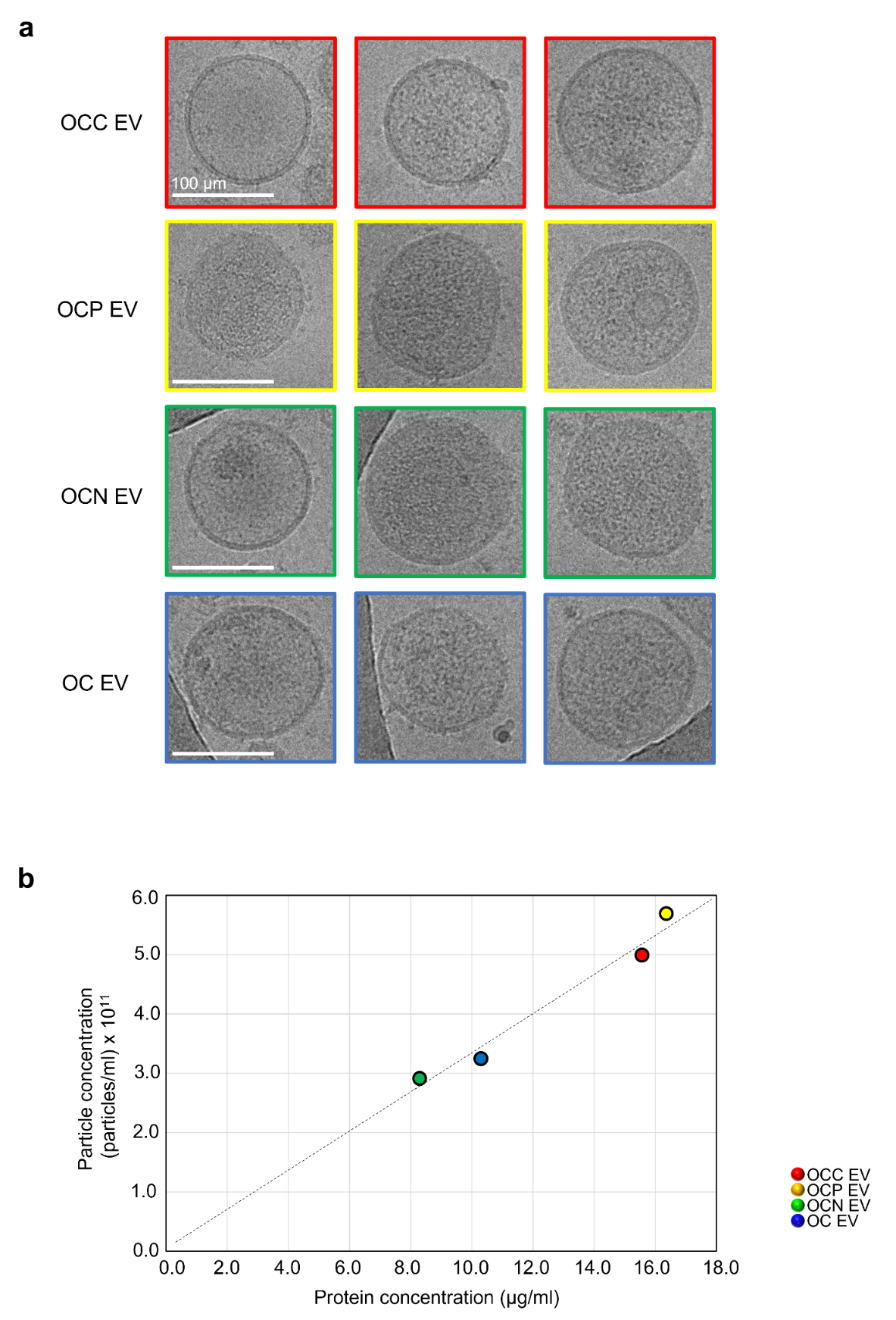


**Supplementary figure 5. (a)** Representative cryo-TEM images showing a standard structure of small EVs from four types of OC. Bars represent 100 μm. **(b)** The correlation between the number of particles and the protein concentration of EV samples derived from each type of OC. The number of particles measured in EV samples with NTA positively correlates with the protein concentration of EV samples.

**
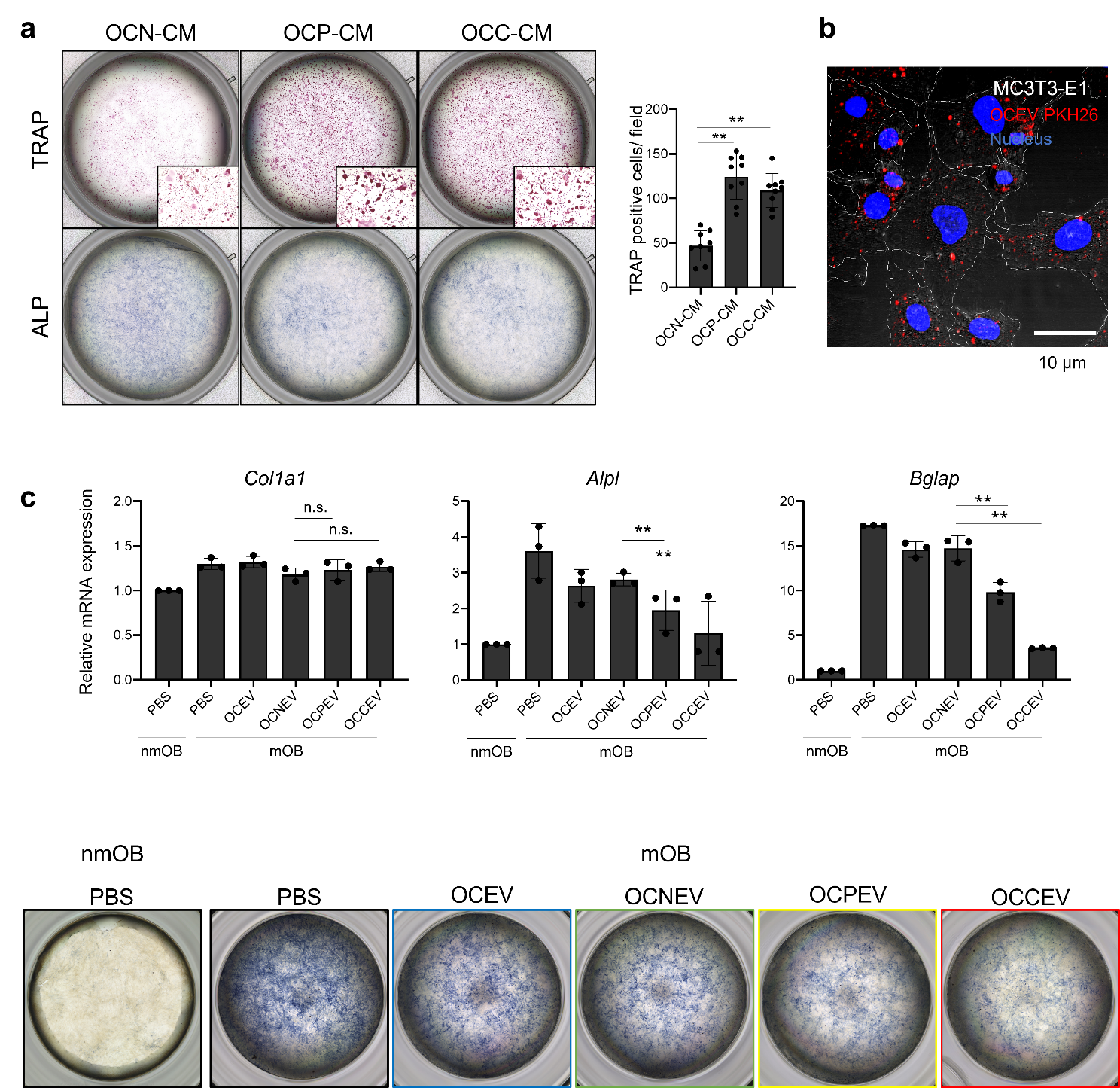
**

**Supplementary figure 6. (a)** TRAP staining (left upper panel) and TRAP positive cell counts (right panel) of mOCs treated with conditioned medium (CM) culturing OCN, OCP, and OCC cells. Error bars represent the SD of the mean (n=9). ***p*<0.01, One-way ANOVA. RAW 264.7 cells were cultured in each CM with RANKL (10 ng/mL) for 5 d. ALP staining of the mOBs treated with CM culturing OCN, OCP, and OCC cells (lower left panel). MC3T3-E1 cells were cultured in CM containing ascorbic acid and β-glycerophosphate for 9 d. **(b)** Representative confocal microscopy images showing the uptake of OC EVs into OB. EVs derived from differentiated RAW 264.7, were labelled with PKH67 red and added to MC3T3-E1cells. Scale bar indicates 10 µm. **(c)** Effects of EV supplementation on the expression of osteoblastic markers in mOBs. The expression levels of *Col1a1*, *Alpl*, and *Bglap* in MC3T3-E1 cells in the presence of ascorbic acid and β-glycerophosphate were measured. Error bars represent SD. **p*<0.05, ***p*<0.01 by Student’s *t*-test. n=3 biological replicates. n.s., no significance (upper panel). ALP staining of mOBs treated with OC EV. MC3T3-E1 cells were cultured with or without ascorbic acid and β-glycerophosphate for 9 days after each OC EV treatment (lower panel).

**
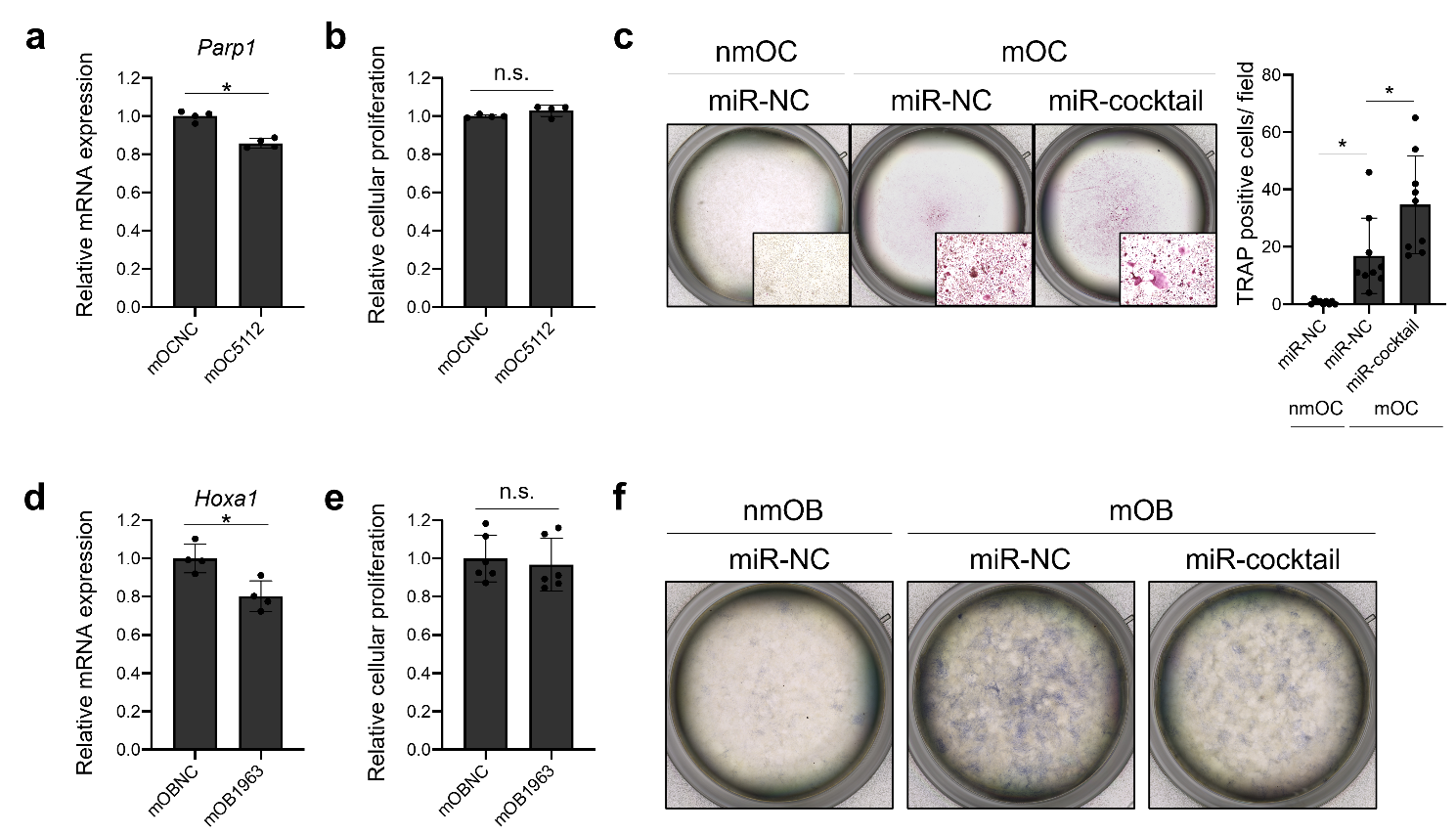
**

**Supplementary figure 7. (a)** Validation test for NGS analysis of miR-5112 targets. qRT-PCR analysis of *Parp1* expressions in RAW 264.7 cells transfected with miR-5112 mimic. NC: negative control. n=3 biological replicates. Error bars represent SD. **p*<0.05, Student’s *t*-test. **(b)** Viability of RAW 264.7 cells after transfection with the miR-5112 mimic. Cell viability was measured using a CCK-8 assay. Error bars represent the SD of the mean (n=4, **p*<0.05, Student’s *t*-test). n.s., no significance. **(c)** TRAP staining (left panel) and TRAP-positive cell counts (right panel) of mOCs transfected with miR-1963 and miR-5112 mimic cocktail. Error bars represent the SD of the mean (n=9). **p*<0.05 by One-way ANOVA. RAW 264.7 cells were cultured with RANKL (10 ng/mL) for 5 days after transient transfection with the miR-mimic. nmOC: non-mOC. NC: negative control. **(d)** Validation test for NGS analysis of miR-1963 targets. qRT-PCR analysis of *Hoxa1* expression in MC3T3-E1 cells transfected with miR-1963 mimic. NC: negative control. n=3 biological replicates. Error bars represent SD. **p*<0.05, Student’s *t*-test. **(e)** Viability of MC3T3-E1 cells after transfection with miR-1963 mimic. Cell viability was measured using a CCK-8 assay. Error bars represent the SD of the mean (n=4, **p*<0.05, Student’s *t*-test). n.s., no significance. **(f)** ALP staining of mOBs transfected with miR-1963 and miR-5112 mimic cocktail. MC3T3-E1 cells were cultured with ascorbic acid and β-glycerophosphate for 9 days after transient transfection with the miR-mimic. nmOB: non-mOB. NC: negative control.


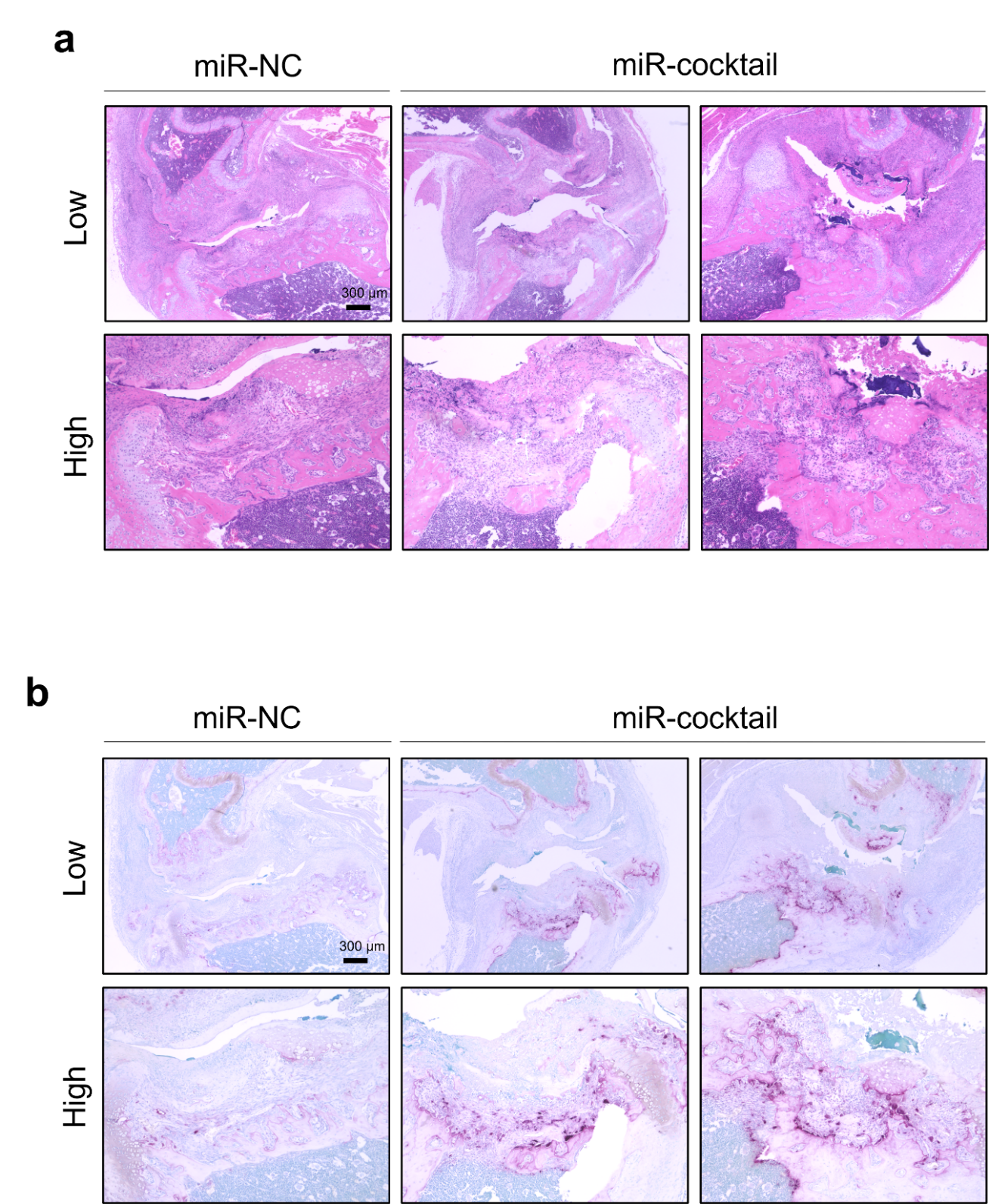


**Supplementary figure 8. (a)** Other images of HE- stained sections of bone metastatic sites in miR mimic-injected mice. All images were obtained with an all-in-one fluorescence microscope using a 10x objective for low- and 20x objective for high-magnification. Scale bar indicates 300 μm. **(b)** Other images of TRAP/ALP-stained sections of bone metastatic sites in miR mimic-injected mice. These images are serial sections from the same murine bone samples as shown in (a). All images were obtained with an all-in-one fluorescence microscope using a 10x objective for low- and 20x objective for high-magnification. Scale bar indicates 300 μm.


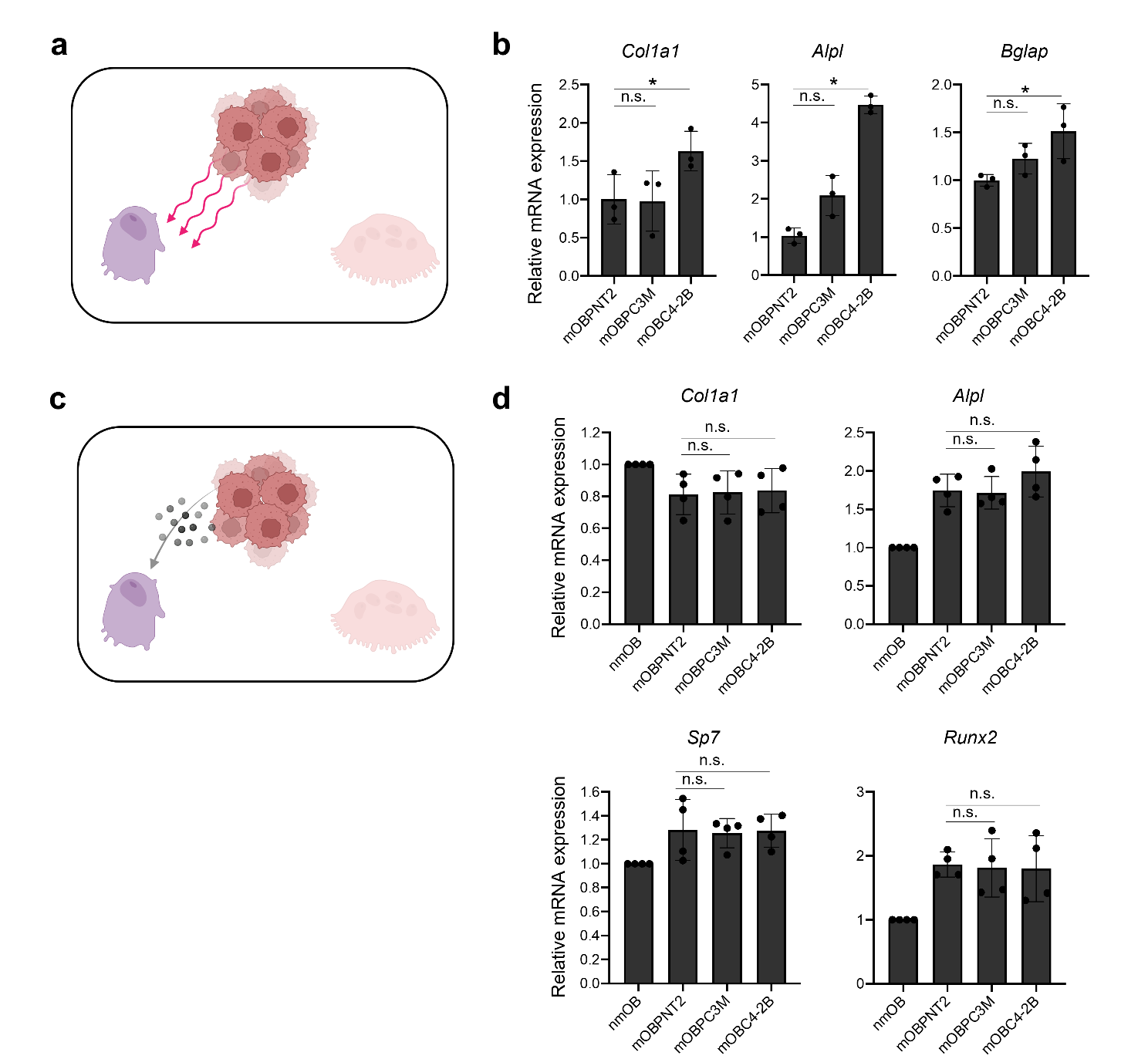


**Supplementary figure 9. (a)** Schematic image of co-culture experiments in mOB with each type of PCa cell (PC3M and C4-2B) and normal epithelial cells (PNT2) as controls. This figure was created using Bio-Render. com. **(b)** Effect of co-culture with PCa on the expression of osteoblastic markers in mOB. The expression levels of *Col1a1*, *Alpl*, and *Bglap* in MC3T3-E1 cells in the presence of ascorbic acid and β-glycerophosphate were measured after 72 h of co-culture. Error bars represent SD. **p*<0.05, Student’s *t*-test. n=3 biological replicates. n.s., no significance. **(c)** Schematic of PCa EV supplementation experiments in mOB treated with PCa-derived EVs. This figure was created using Bio-Render.com. **(d)** Effect of PCa EV supplementation on the expression of osteoblastic markers in mOBs. The expression levels of *Col1a1*, *Alpl*, *Sp7*, *Runx2* in MC3T3-E1 cells in the presence of ascorbic acid and β-glycerophosphate were measured. Error bars represent SD. **p*<0.05, ***p*<0.01 by Student’s *t*-test. n=four biological replicates. n.s., no significance.

**Supplementary tables**

**Supplementary table 1.** **List of oligonucleotides used in this study and supplier details.**

**Supplementary table 2. Primer sequences for qRT-PCR analyses.**

**Supplementary table 3. Quantitative ImageJ analysis of TRAP staining in OCs treated with denosumab.**

**Supplementary table 4. Top signaling pathways enriched in CAOCs from the gene set enrichment analysis (GSEA).**

**

**

**Supplementary table 5. Data of four types of OC-derived EV samples.**

**Supplementary table 6. A list and average read counts of miRNAs enriched in CAOC EVs.**

**

**

**Supplementary materials and methods**

**Quantitative ImageJ analysis of TRAP staining in OCs** **treated with denosumab.**

To assess the extent of damage induced by denosumab in the four types of OCs, TRAP staining images were analyzed using the ImageJ software. ImageJ is a widely used, open-source Java-based image processing program that supports 8-bit, 16-bit, and 32-bit color images. Each image underwent binarization followed by particle analysis. The software measured the area of the target objects, and the total area of TRAP-positive regions was calculated to determine the extent of damage. Objects smaller than 50 μm² were considered noise and excluded from the analysis.
